# A novel multi-objective dynamic flexible job shop scheduling algorithm using reinforced learning based black widow spider algorithm

**DOI:** 10.1371/journal.pone.0347108

**Published:** 2026-04-20

**Authors:** Kashif Akram, Muhammad Usman Bhutta, Shahid Ikramullah Butt, Muhammad Rizwan, Muhammad Salman Khan, Mushtaq Khan, Alamzeb Khan

**Affiliations:** 1 School of Mechanical & Manufacturing Engineering (SMME), Campus H-12, National University of Sciences & Technology (NUST), Islamabad, Pakistan; 2 Anglia Ruskin University (ARU), Bishop Hall Ln, Chelmsford CM1 1SQ, United Kingdom; 3 Mechanical Engineering Department, College of Engineering, Prince Mohammad Bin Fahd University, Al Khobar, Saudi Arabia; Aalto University, FINLAND

## Abstract

In today’s fast-paced manufacturing environments, solving flexible job shop scheduling problem (FJSP) has become essential due to swift design-to-manufacturing-to-consumer cycle and frequent disruptive events like new job arrivals. This study proposes a novel reinforcement learning based black widow spider algorithm (BWSA-RL) to address the multi-objective dynamic flexible job shop scheduling problem (MODFJSP). The algorithm utilizes a hybrid reinforcement learning framework for dynamic adjustment of procreation and mutation rates of BWSA-RL. The switch between SARSA and Q-learning is achieved through a novel conversion operator based on sparsity of Q-tables. To enhance Pareto front diversity, a novel hybrid crowding distance metric (HCD) is introduced. Additionally, a rescheduling-heuristic is proposed to accommodate new job arrivals. A comprehensive experimental regime was applied to validate the proposed novelties against 30 benchmark instances. Mathematical model was validated with mixed integer linear programming (MILP). The conversion condition operator and the HCD metric were benchmarked against two other approaches, demonstrating their effectiveness in balancing exploration and exploitation while maintaining solution diversity. BWSA-RL was benchmarked against four state-of-the-art algorithms, outperforming them in 83.3% of the instances. BWSA-RL demonstrated its potential as a robust approach for MODFJSP, balancing energy efficiency and operational goals like makespan, due-date conformance and schedule stability.

## 1. Introduction

Efficient production scheduling is essential for meeting the demands of modern-day manufacturing, characterized by increased variability, reduced batch sizes, and extensive customizations [1]. The NP-hard flexible job shop scheduling problem (FJSP) is a widely recognized challenge in current manufacturing industries, such as semiconductor manufacturing, automobile parts manufacturing, and transportation logistics [2]. The dynamic flexible job shop scheduling problem (DFJSP) involves assigning and sequencing operations across multiple machines while adapting to disruptions like machine breakdowns and priority changes to maintain manufacturing efficiency and stability. Additionally, real-world scheduling often requires optimizing multiple conflicting objectives, leading to the multi-objective dynamic flexible job shop scheduling problem (MODFJSP). Complexity of MODFJSP demands advanced algorithms to balance competing goals [3], typically addressed through weighted-objective methods or Pareto-based approaches, the latter offering production managers greater flexibility in selecting optimal scheduling solutions [3,4].

Since FJSP’s introduction by Brucker and Schlie [5], it has attracted significant research interest due to its application potential in real-world manufacturing systems. The complexity of FJSP makes exact methods impractical for solving large-scale instances. Metaheuristic algorithms have emerged as a preferred solution approach, balancing solution quality with computational efficiency. Researchers have explored various metaheuristic approaches, including the multi-objective discrete Jaya algorithm [6], non-dominated sorting genetic algorithm (NSGA-II) [7], and game-theory-based strategies [8] to enhance solution quality. Additionally, the improved spider monkey optimization [9] and hybrid shuffled frog leaping algorithm [10], have been proposed to improve scheduling efficiency in dynamic environments.

Energy consumption has become a key consideration in scheduling, with recent studies incorporating energy efficiency into MODFJSP objectives. Various optimization techniques, such as memetic NSGA-II [11], enhanced NSGA-II [12], and improved grey wolf optimization [13], have been developed to minimize makespan while optimizing energy consumption. Bi-population evolutionary algorithms [14] and iterative tabu search methods [15] have further refined energy-efficient scheduling by balancing completion times and real-time energy tariffs. Other approaches, such as knowledge-based evolutionary algorithms [16] and genetic programming based hyper-heuristics [17], leverage machine learning principles to enhance scheduling efficiency while reducing energy consumption. These studies emphasize the need for multi-objective optimization strategies that consider both production efficiency and environmental-economical sustainability.

The integration of reinforcement learning (RL) into metaheuristic algorithms has further advanced FJSP solutions, allowing for adaptive decision-making in dynamic environments. Deep Q-learning [18], proximal policy optimization [19], and artificial bee colony-based RL approaches [20] have demonstrated improved scheduling flexibility and performance. Reinforcement learning models, including graph neural networks [21] and graph reinforcement learning [22], have been applied to optimize sequencing and machine assignments. Hybrid approaches that combine RL with NSGA-II [23], and transformer networks [24] have also been explored to enhance scheduling under uncertainties such as machine breakdowns and variable processing times. These methods highlight the potential of RL-driven optimization frameworks in handling complex scheduling challenges.

Job priority consideration in FJSP remains a relatively underexplored area, despite its importance in real-world applications where jobs differ in urgency and penalties for delays. Researchers have developed priority-based scheduling models using heuristics such as critical ratio and earliest start time, particularly in sectors like automobile repair and just-in-time manufacturing [25]. Hybrid metaheuristic approaches, including tabu-variable neighborhood search [26] and quantum annealing-based algorithms [27], have been introduced to optimize job sequencing while considering priority constraints. Other studies have incorporated outsourcing and weighted penalty mechanisms [28] to balance overdue days and delay penalties. These contributions underscore the need for advanced scheduling models that integrate job priority levels to enhance real-world applicability.

Existing FJSP research largely assumes equal job priority, overlooking realistic variations and their impact on scheduling solutions. Additionally, there is a lack of heuristics designed to reduce instability from new job insertions while considering priority constraints. Existing RL implementations rely on a rigid SARSA-to-Q-learning switch, which limits adaptability and efficiency, highlighting the need for a dynamic switching mechanism. Moreover, Pareto-based solutions often use Euclidean or Hamming distance metrics, which can lead to inaccuracies due to scaling effects, necessitating an improved crowding distance metric. To address these gaps, this study proposes a Pareto-optimal black widow spider algorithm (BWSA) for solving MODFJSP while optimizing makespan, total energy consumption, average due-date penalty, and schedule instability. RL is incorporated to dynamically adjust procreation and mutation rates, while a rescheduling heuristic is developed to minimize instability caused by new job insertions. The main contributions of this study are summarized as follows.

1. A MILP model that integrates optimization objectives and accommodates three levels of job priorities.
2. A novel hybrid implementation of BWSA and RL for solving MODFJSP.
3. A novel conversion condition operator to control switching from SARSA to Q-learning.
4. A novel hybrid cosine distance metric to promote diversity in evolutionary populations.
5. A novel rescheduling heuristic to manage new job insertion.

**Fig 1:**
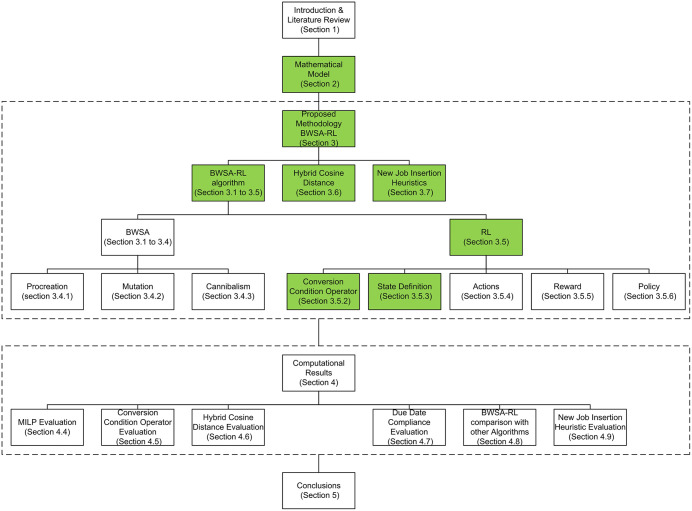
Research methodology and overall organization of the proposed study.

The reset of the paper is organized as: section 1 provides introduction, literature review and research gap, section 2 provides mathematical model, section 3 delves into the details of the proposed algorithm, section 4 presents computational results and section 5 provides conclusions. The research methodology is explained in Fig 1, the green highlighted boxes are the novelties proposed in this research paper.

## 2. Problem formulation

### 2.1. Problem model

An n×m MODFJSP can be defined as a system of n jobs J, where $J=\{J_{\text{1}},\;J_{\text{2}},\;J_{\text{3}},\ldots ,\;J_{n}\}$, which are to be process on m machines M, where $M=\{M_{\text{1}},\;M_{\text{2}},\;M_{\text{3}},\;\ldots ,\;M_{m}\}$. Each job $J_{i}$ consist of $n_{i}$ operations $O_{i,j}$, where $O_{i,j}=\{O_{i,\text{1}},\;O_{i,\text{2}},\;O_{i,\text{3}},\;\ldots ,\;O_{i,n_{i}}\}$. Every operation $O_{i,j}$ can be performed on the given set of candidate machines $M_{i,j}$, where $M_{i,j}$ = {machines capable of performing operation $O_{i,j}$}.

This research divides scheduling into stages: Stage 1 focuses on optimizing makespan (MK), total energy consumption (TEC), and average due-date penalty (ADP), forming a Pareto front of elite solutions for managers to select from. Stage 2 and beyond handle rescheduling due to disruptions, introducing an additional objective, instability (INS), which measures deviations from the previous schedule. A 4 × 3 sample problem is presented in Table 1, with its energy requirements detailed in Table 2. If a job’s operation cannot be performed on a specific machine, it is marked with an infinity sign in Table 1. In the next section a formal mathematical model of the proposed problem is presented.

**Table 1:** A 4x3 sample problem.

Job	Priority	Operation 1			Operation 2			Operation 3		
		M1	M2	M3	M1	M2	M3	M1	M2	M3
J1	Urgent	4	4	4	∞	3	3	∞	4	∞
J2	Normal	2	∞	∞	2	2	4	∞	∞	2
J3	Low	∞	2	2	4	4	∞	4	2	4
J4	Normal	2	∞	∞	∞	3	2	4	4	4

**Table 2:** Estimated process, idle and on/off energies of the sample problem.

Energy type	M1	M2	M3
Processing energy	0.76	0.83	0.64
Idling energy	0.22	0.21	0.24
On/Off energy	0.21	0.04	0.21

### 2.2. Mathematical model

In this section, mathematical model is proposed which integrates four objective functions and relevant constraints. The required indices, parameters, and variables are defined prior to introducing the mathematical model.

- **Indices**
  - i:- jobs iteration index, $i=\text{1},\;\text{2},\;\text{3},\ldots ,\;N_{s}$
  - j:- operations iteration index, $j=\text{1},\;\text{2},\;\text{3},\ldots ,\;n_{i,s}$
  - k:- machine iteration index, k=1, 2, 3,…, m
  - p:- indices representing the operational positions on machine k, $p=\text{1},\;\text{2},\;\text{3},\ldots ,\;d_{k,s}$
  - s:- indices are used to define stages, with s=1, 2, …∞ where s = 1 denotes the initial stage and s≥2 corresponds to rescheduling stages

- Parameters
  - $N_{s}$:- total jobs to be scheduled during stage s
  - $n_{i,s}$:- total operations of job i during stage s
  - m:- total available machines
  - $d_{k,s}$:- the total number of operations assigned to machine *k* in stage *s*
  - $C_{k,s}$:- end time of last operation on machine k during stage s
  - $t_{i,j,k}$:- processing time on machine k for operation $O_{i,j}$
  - $\overset{P}{\underset{k}{E}}$:- energy consumption rate for processing an operation on machine k
  - $\overset{I}{\underset{k}{E}}$:- idling energy consumption rate for keeping machine k in standby for next job
  - $\overset{S}{\underset{k}{E}}$:- energy needed for one complete On/Off cycle of machine 𝑘
  - $B_{i,j,s}$:- operation $O_{i,j}$ start time during stage s
  - $F_{i,j,s}$:- operation $O_{i,j}$ finish time during stage s
  - $S_{k,p,s}$:- start time for an operation assigned to machine 𝑘 in position 𝑝 at stage 𝑠
  - $C_{k,p,s}$:- completion time for an operation assigned to machine 𝑘 in position 𝑝 at stage 𝑠
  - $w_{H}$:- weight assigned to high priority jobs for ADP calculation
  - $w_{N}$:- weight assigned to normal priority jobs for ADP calculation
  - $w_{L}$:- weight assigned to low priority jobs for ADP calculation
  - $C_{i}$:- completion time of i^th^ job
  - $D_{i}$:- due-date of i^th^ job
  - $Q_{i}$:- release time of the first operation of job 𝑖
  - M:- An arbitrarily large number
- Variable
  -
$W_{i,j,k,s}=\left\{ \begin{array}{c} @l\text{1}\;,\;if\;operation\;O_{i,j}is\;processed\;on\;machine\;k\;at\;stage\;s\\ \text{0}\;,\;otherwise \end{array}\right.$

  -
$X_{i,j,k,p,s}=\left\{ \begin{array}{c} @l\text{1}\;,\;if\;operation\;O_{i,j}is\;processed\;on\;machine\;k\;at\;position\;p\;at\;stage\;s\\ \text{0}\;,\;otherwise \end{array}\right.$

  -
$Y_{i,j,s}=\left\{ \begin{array}{c} @l\text{1}\;,\;if\;procesing\;machine\;for\;operation\;O_{i,j}\;is\;same\;at\;both\;stages\;s\;and\;s-\text{1}\\ \text{0},\;otherwise \end{array}\right.$

  -
$Z_{i,j,s}=\left\{ \begin{array}{c} @l\text{1}\;,\;if\;start\;time\;of\;operation\;O_{i,j}\;is\;same\;at\;both\;stages\;s\;and\;s-\text{1}\\ \text{0},\;otherwise \end{array}\right.$

  -
$\Phi_{i}=\left\{ \begin{array}{c} @l\text{1}\;,\;if\;priority\;of\;job\;J_{i}=High\\ \text{0},\;otherwise \end{array}\right.$

  -
$\Psi_{i}=\left\{ \begin{array}{c} @l\text{1}\;,\;if\;priority\;of\;job\;J_{i}=Normal\\ \text{0},\;otherwise \end{array}\right.$

  -
$\Omega_{i}=\left\{ \begin{array}{c} @l\text{1}\;,\;if\;priority\;of\;job\;J_{i}=Low\\ \text{0},\;otherwise \end{array}\right.$

**Objective functions;**

(1) Minimize makespan (MK)

$$f_{\text{1}}=\text{max}\left\{C_{k,s}\right\}\;$$ (1)

(2) Minimize total energy consumption (TEC)

$$f_{\text{2}}=\sum_{i=\text{1}}^{N_{s}}\sum_{j=\text{1}}^{n_{i,s}}t_{i,j,k}\overset{P}{\underset{k}{E}}W_{i,j,k,s}+\sum_{k=\text{1}}^{m}\sum_{p=\text{1}}^{d_{k,s}-\text{1}}\text{min}\left\{\overset{I}{\underset{k}{E}}\left(S_{k,(p+\text{1}),s}-C_{k,p,s}\right),\;\overset{S}{\underset{k}{E}}\right\}\;$$ (2)

(3) Minimize average due date penalty (ADP)

$$f_{\text{3}}=\frac{\text{1}}{N_{s}}\sum_{i=\text{1}}^{N_{s}}\left|C_{i}-D_{i}\right|\left(w_{H}\Phi_{i}+w_{N}\Psi_{i}+w_{L}\Omega_{i}\right)\;$$ (3)

(4) Minimize instability (INS)

$$f_{\text{4}}=\frac{\sum_{i=\text{1}}^{N_{s}}\sum_{j=\text{1}}^{n_{i,s}}\{\text{0}.\text{5}{(\text{1}-Y}_{i,j,s})+\text{0}.\text{5}{(\text{1}-Z}_{i,j,s})\}}{\sum_{i=\text{1}}^{N_{s}}n_{i,s}}\;$$ (4)

The objective functions are constraint by


$$\sum_{k=\text{1}}^{m}W_{i,j,k,s}=\text{1},\;i=\text{1},\text{2},\ldots ,N_{s},\;j=\text{1},\text{2},\ldots ,n_{i,s},\;s=\text{1},\text{2},\ldots ,\infty \;$$ (5)

$$S_{k,(p+\text{1}),s-}C_{k,p,s}\geq \text{0},\;k=\text{1},\text{2},\ldots ,m,\;p=\text{1},\text{2},\ldots ,d_{k,s}-\text{1},\;s=\text{1},\text{2},\ldots ,\infty \;$$ (6)

$$B_{i,(j+\text{1}),s-}F_{i,j,s}\geq \text{0},\;i=\text{1},\text{2},\ldots ,N_{s},\;j=\text{1},\text{2},\ldots ,n_{i,s}-\text{1},\;s=\text{1},\text{2},\ldots ,\infty \;$$ (7)

Equation (5) restricts loading of operation $O_{i,j}$ more than once, equation (6) puts restriction of simultaneous loading of more than one operation on single machine, inherent precedence constraints are ensured through equation (7).


$$B_{i,j,s}\leq S_{k,p,s}+M\left(\text{1}-X_{i,j,k,p,s}\right),\;i=\text{1},\text{2},\ldots ,N_{s},\;j=\text{1},\text{2},\ldots ,n_{i,s},\;k=\text{1},\text{2},\ldots ,m,\;p=\text{1},\text{2},\ldots ,d_{k,s}\;s=\text{1},\text{2},\ldots ,\infty \;$$ (8)

$$B_{i,j,s}\geq S_{k,p,s}-M(\text{1}-X_{i,j,k,p,s}),\;i=\text{1},\text{2},\ldots ,N_{s},\;j=\text{1},\text{2},\ldots ,n_{i,s},\;k=\text{1},\text{2},\ldots ,m,\;p=\text{1},\text{2},\ldots ,d_{k,s}\;s=\text{1},\text{2},\ldots ,\infty \;$$ (9)

$$F_{i,j,s}\leq C_{k,p,s}+M(\text{1}-X_{i,j,k,p,s}),\;i=\text{1},\text{2},\ldots ,N_{s},\;j=\text{1},\text{2},\ldots ,n_{i,s},\;k=\text{1},\text{2},\ldots ,m,\;p=\text{1},\text{2},\ldots ,d_{k,s}\;s=\text{1},\text{2},\ldots ,\infty \;$$ (10)

$$F_{i,j,s}\geq C_{k,p,s}-M(\text{1}-X_{i,j,k,p,s}),\;i=\text{1},\text{2},\ldots ,N_{s},\;j=\text{1},\text{2},\ldots ,n_{i,\;s},\;k=\text{1},\text{2},\ldots ,m,\;p=\text{1},\text{2},\ldots ,d_{k,s}\;s=\text{1},\text{2},\ldots ,\infty \;$$ (11)

Equations (8–9) and (10–11) links start and finish times of operation $O_{i,j}$ with machine k respectively.


$$B_{i,j,s}\geq Q_{i},\;i=\text{1},\text{2},\ldots ,N_{s},\;j=\text{1},\;s=\text{1},\text{2},\ldots ,\infty \;$$ (12)

Equation (12) ensures that the first operation of any job is scheduled no earlier than its arrival time $Q_{i}$. For the initial scheduling stage, $Q_{i}$ is zero, while for subsequent rescheduling stages, $Q_{i}$ equals the dynamic event time $T_{e}$.


$$\sum_{i=\text{1}}^{N_{s}}\sum_{j=\text{1}}^{n_{i}}X_{i,j,k,p,s}=\text{1},\;k=\text{1},\text{2},\ldots ,m,\;p=\text{1},\text{2},\ldots ,d_{k,s},\;s=\text{1},\text{2},\ldots ,\infty$$ (13)

Equation (13) restricts assigning of more than one operations in overlapping timeslots on machine k. Following are the assumptions applied on this study.

(1) Pre-emption is not allowed once an operation begins on a machine.
(2) Processing times for all job operations are deterministic, including setup, loading, and unloading times.
(3) Machine energy consumption remains constant throughout scheduling.

## 3. Proposed reinforcement learning based Black widow spider algorithm (BWSA-RL)

### 3.1. Overview of BWSA-RL framework

This section introduces the proposed BWSA-RL for solving the MODFJSP. The proposed approach integrates a population-based evolutionary optimization framework with a reinforcement learning (RL) controller that adaptively regulates key evolutionary parameters during the search process.

The core optimization engine of BWSA-RL is derived from the black widow spider algorithm (BWSA), which evolves a population of candidate schedules through biologically inspired operators such as procreation, mutation, and cannibalism. In contrast to conventional BWSA variants that rely on static parameter settings, BWSA-RL introduces an adaptive control mechanism in which RL dynamically adjusts the procreation rate $R_{p}$ and mutation rate $R_{m}$ based on the observed search performance. This adaptive strategy enables the algorithm to respond effectively to different optimization phases and problem scales. In the following paragraph an overview of the working of the proposed algorithm is provided.

**Fig 2:**
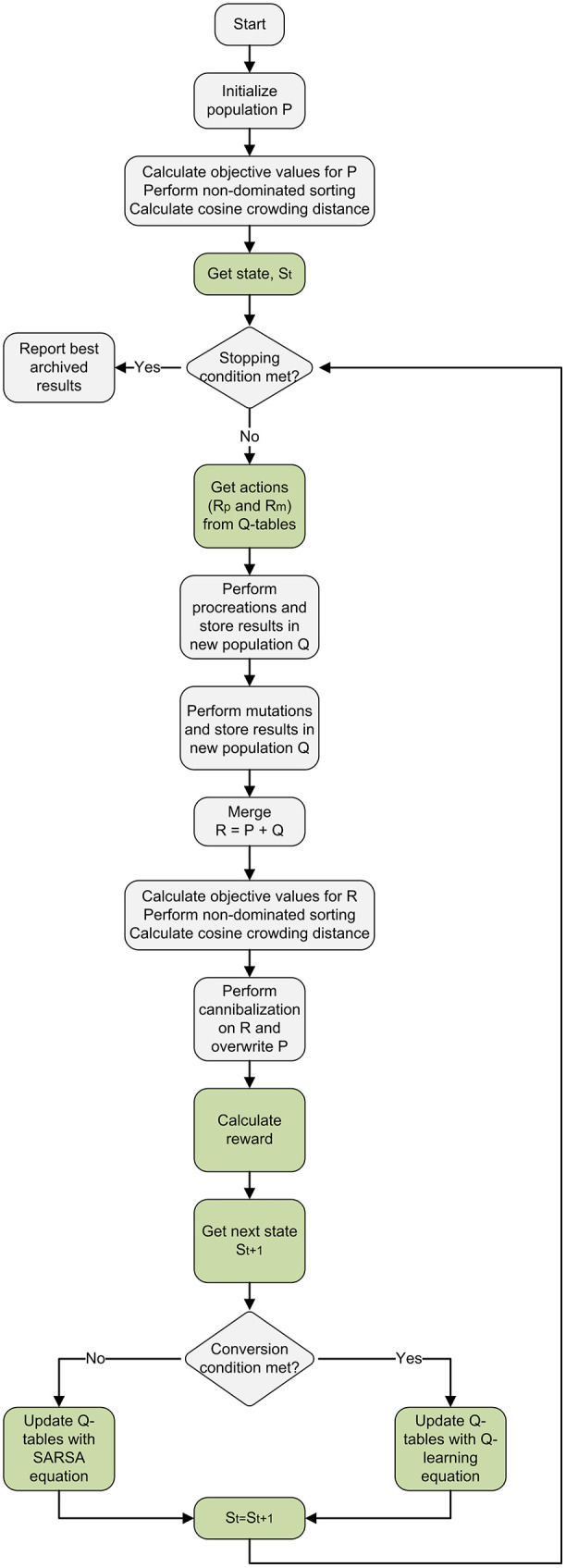
BWSA-RL framework illustrating the algorithmic control flow and integration of the reinforcement learning module.

The Fig 2 illustrates the overall workflow of the proposed BWSA-RL framework. The algorithm starts with generation of an initial population P of feasible solutions using a hybrid initialization strategy, after that this initial population is evaluated and sorted in Pareto fronts through non-dominated sorting algorithm. The Q-tables for $R_{p}$ and $R_{m}$ are initialized with zeros, the initial state $S_{t}$ is determined and the values of $R_{p}$ and $R_{m}$ are extracted from the action table as per RL policy π. Procreation and mutation are performed using RL suggested $R_{p}$ and $R_{m}$, and newly generated offspring solutions are kept in a container population Q. After completion of these evolutionary processes, the population P and Q are merged into a new population R. Population R is evaluated, sorted in Pareto fronts and hybrid crowding distance metric is calculated. Then cannibalism, an elitist selection mechanism, is performed on R until its size reduces to the original size of population P, then all the members of P are replaced with R. At the end of each generation, the reward $R_{t+\text{1}}$ is calculated and the next state $S_{t+\text{1}}$ is determined as per policy π and relevant update of Q-tables is performed by either SARSA or Q-learning. These processes keep on repeating until the stopping conditions are met, i.e., the maximum number of generations is reached, or no improvement is seen in the elite Pareto front over the last 20 generations. The time and space complexity of BWSA-RL is $O(\eta^{\text{2}})$ and O(η) respectively where η is the total number of operations to be scheduled.

### 3.2. Encoding and decoding

**Fig 3:**
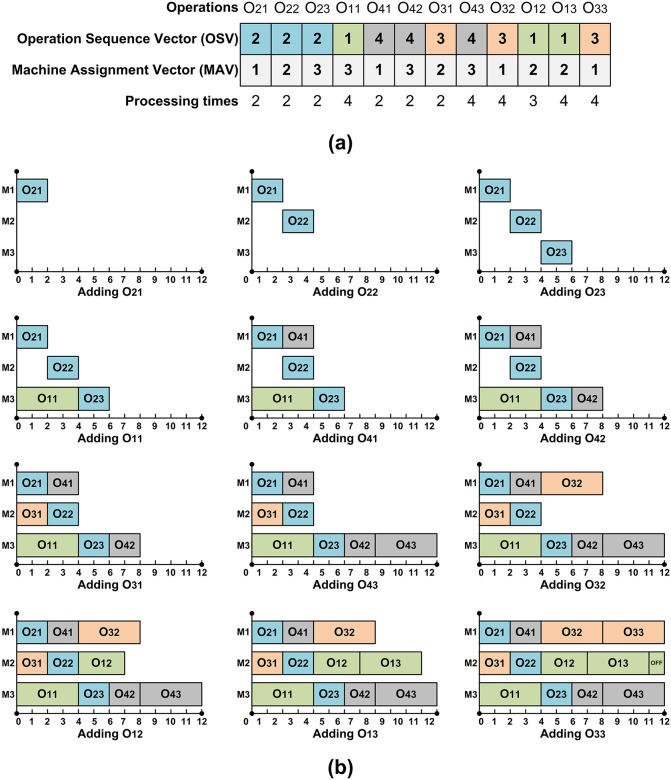
Encoding and decoding of OSV and MAV. a) an encoded solution, b) step by step decoding using G&T algorithm.

Two vector encoding method [29] is adopted for this study, process sequence is stored in one vector named operation sequence vector (OSV) and machine allocation is stored in another vector named machine assignment vector (MAV). The size of both OSV and MAV is equal to the total operations to be scheduled in the current stage. The operation precedence constraint is embedded in OSV, and corresponding machine codes are stored in MAV. Fig 3a shows a two-vector encoding for an optimal solution to the sample problem presented in Table 1. Reading from left to right, the first 2 represent the first operation $O_{\text{2}\text{1}}$ of job 2 which is to be performed on machine 1, similarly second appearance of 2 represents operation $O_{\text{2}\text{2}}$ of job 2 which is to be processed on machine 2.

To decode the encoded solutions, G&T [30,31] algorithm is employed. This algorithm generates solutions in the active region of solution space which contains optimal solutions [31]. Step by step decoding is explained in Fig 3b, by reading OSV and MAV from left to right first operation $O_{\text{2}\text{1}}$ is loaded on machine 1 for 2 processing units of time. Then the second operation $O_{\text{2}\text{2}}$ is loaded on machine 2 for 2 units of time, to maintain precedence constraint the starting of this operation must be on or after completion time of operation $O_{\text{2}\text{1}}$. In this way all the subsequent operations are scheduled on the machines.

### 3.3. Population initialization

The quality and diversity of the initial population significantly impact the convergence of evolutionary algorithms to optimal or near-optimal solutions [2]. This study employs a set of initialization rules, each targeting a specific objective. For makespan minimization, three rules are used: the most operations remaining rule [32], the most work remaining rule [33], and the global minimum time rule [34]. Total energy consumption is optimized using the minimum energy rule and the minimum energy load rule [35]. Average due-date penalty is addressed through a priority-based rule that encodes jobs in high-to-low priority order. Schedule instability is reduced using a stability-considered rule that assigns operations to the same machines as in the previous schedule. Additionally, random initialization for operation sequence vectors (OSV) and machine assignment vectors (MAV) are incorporated to enhance population diversity.

### 3.4. Evolutionary operators of BWSA-RL

#### 3.4.1. Procreation.

The goal of precreation is to create new individuals through mating of parent spiders from the current population. The total numbers of mattings $N_{p}$ are controlled through the parameter $R_{p}$. The expression for $N_{p}$ is given in equation 14.


$$N_{p}=P_{size}\times R_{p}\;$$ (14)

**Fig 4:**
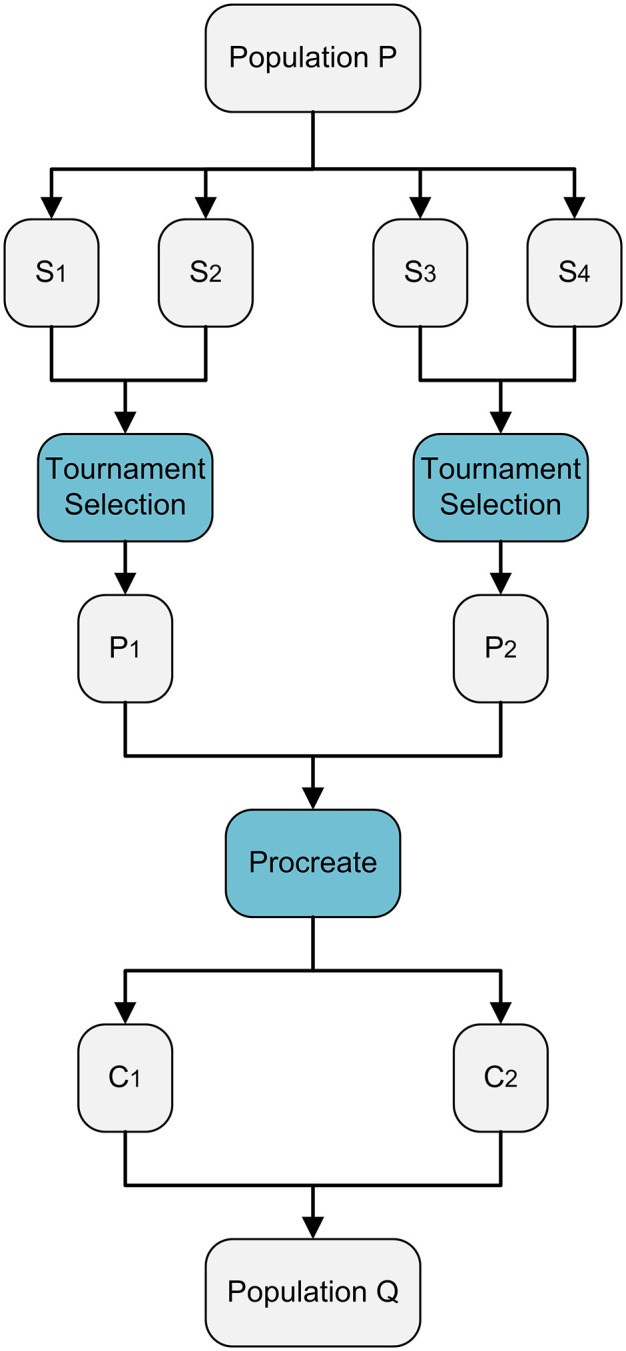
Procreation mechanism for parent selection based on a tournament selection strategy.

Where $P_{size}$ is the total individuals in P. Higher the value of $R_{p}$ more crossover operations will be performed and vice versa. Parent selection is performed using tournament selection. Two random individuals from the population are chosen, and the stronger solution is selected as P₁ based on the rank. If both have the same rank, crowding distance (section 3.6) acts as a tiebreaker, favoring the higher value. The process is repeated to select the second parent, P₂. Then both parents are subjected to procreation and produce two children solutions which are stored in Q, this mechanism is explained in Fig 4.

**Fig 5:**
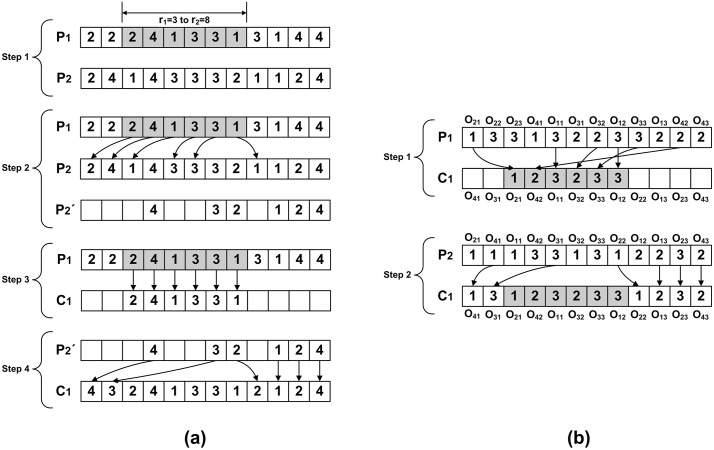
Procreation process for generating offspring spiders from parents P1 and P2:(a) OSV generation steps, and (b) MAV generation steps.

The procreation is completed in two phases, in first phase the OSVs and in second phase MAVs of both parents pass genes to offspring. The OSV of child spider is generated in following four steps, explained graphically in Fig 5a, the particulars of these steps are given below.

Step 1: two random number r1 and r2 are generated such that 1≤r1≤q−1 and r1<r2≤q, where q is the total elements in OSV. For example, r1=3 and r2=8, refer to step one of Fig 5a.
Step 2: for every operation in $P_{\text{1}}$ that falls between r1 and r2, its corresponding operation in $P_{\text{2}}$ is identified and removed to create ${P_{\text{2}}}^{\prime }$. For instance, in $P_{\text{1}}$, job number 2 is found at the third position. The first appearance of job 2 in the OSV of $P_{\text{2}}$ is at the first position, so this entry is removed from $P_{\text{2}}$.
Step 3: the genes between r1 and r2 are copied in child solution $C_{\text{1}}$.
Step 4: the genes in ${P_{\text{2}}}^{\prime }$ are copied, in order of appearance, in empty locations of $C_{\text{1}}$ to form complete OSV of child $C_{\text{1}}$.

The MAV of child spider is generated in following two steps, explained graphically in Fig 5b, the particulars of these steps are given below.

Step 1: the machine assignment between, previously generated, r1 and r2 of $C_{\text{1}}$ are copied from MAV of $P_{\text{1}}$ as shown in Fig 5b. For example, the third position in OSV of $C_{\text{1}}$ represents operation $O_{\text{2}\text{1}}$, therefore machine assignment of $O_{\text{2}\text{1}}$ from $P_{\text{1}}$ is copied on this location.
Step 2: the remaining machine assignments are copied from the MAV of $P_{\text{2}}$. For example, the first location of OSV of $C_{\text{1}}$ represents operation $O_{\text{4}\text{1}}$ and therefore its assignment is copied from second position from MAV of $P_{\text{2}}$.

The procreation technique ensures feasible child solutions without needing a repair mechanism. The second offspring $C_{\text{2}}$ is generated by swapping parents and following the same procedure. After procreation, the stronger parent is designated as mother and the other as father, the weaker father is removed (cannibalized) from the population. This approach enhances diversity and promotes the inheritance of stronger characteristics in the BWSA-RL algorithm.

#### 3.4.2. Mutation.

The purpose of mutation is to create diversity through the arbitrary introduction of characteristics that may not be present in the current population. The total number of mutations $N_{m}$ are controlled through parameter $R_{m}$, the expression for $N_{m}$ is given in equation 15.


$$N_{m}=P_{size}\times R_{m}$$ (15)

**Fig 6:**
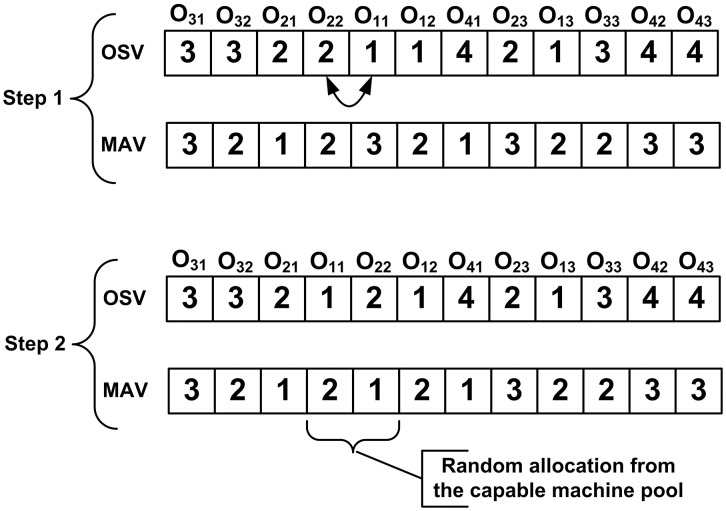
Mutation operator consisting of (a) operation swapping and (b) random reassignment of machines.

Where $P_{size}$ is the total members in P. To perform mutation, a solution is randomly selected from P, the mutation is completed in two stages as depicted in Fig 6.

Step 1: a random value r is selected from the interval [1,q−1], where q represents the total gene count in OSV or MAV.
Step 2: the operations at positions r and r + 1 in OSV are swapped, and a random machine is assigned to each in MAV.

This proposed mutation strategy of BWSA-RL always results in feasible solutions therefore repair strategy is not required.

#### 3.4.3. Cannibalism and population update.

**Fig 7:**
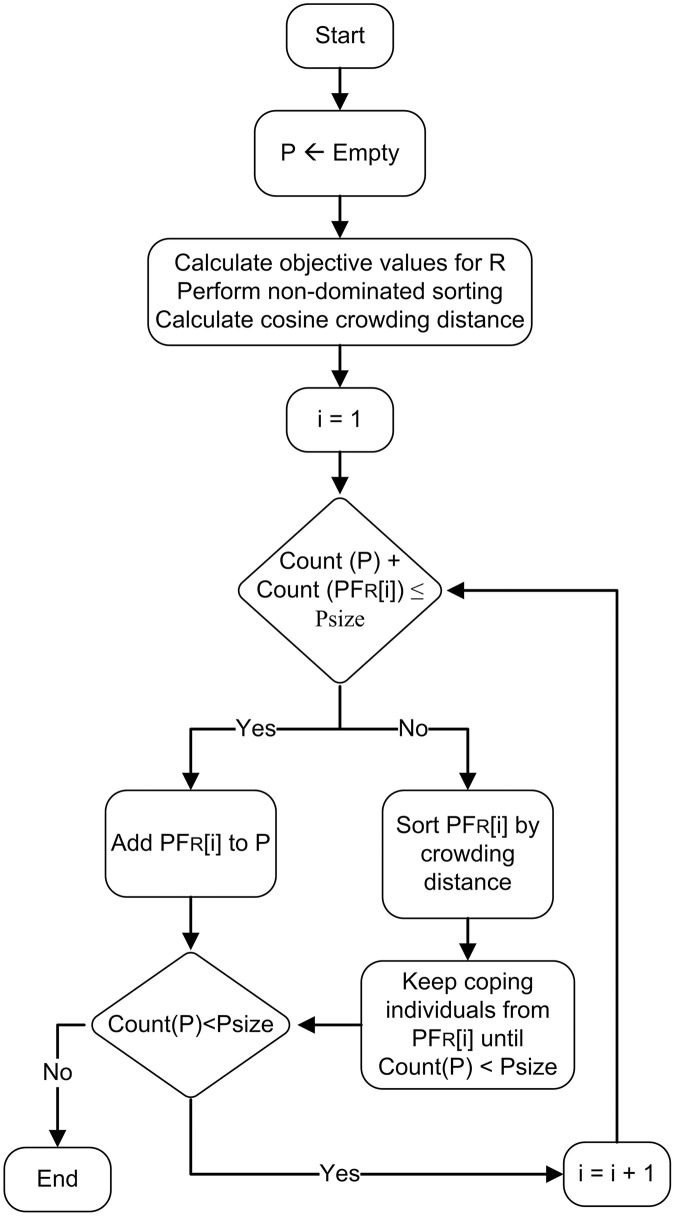
Cannibalization process illustrating the elimination of weaker solution spiders from the population.

Cannibalization involves removing weaker individuals from the population R, ensuring that only stronger and more diverse solutions are passed on to future generations. The population R is divided into Pareto fronts, and the hybrid crowding distance for each member is subsequently calculated. The complete process of cannibalization is summarized in the following steps and a flowchart of the process is shown in Fig 7.

Step 1: evaluate population R, identify Pareto fronts through non-dominated sorting, calculate hybrid crowding distance, and delete all members of population P.
Step 2: set iteration variable i=1.
Step 3: if the combined number of members in population P and the $i^{th}$ Pareto front of R does not exceed the maximum population size, go to step 4; otherwise, jump to step 5.
Step 4: copy $i^{th}$ Pareto front of R to population P. go to step 6.
Step 5: sort the $i^{th}$ Pareto front by crowding distance in descending order and sequentially copy individuals to P until the count of members reaches $P_{size}$.
Step 6: check if the number of members in P is equal to maximum population size, then go to step 7 else set i = i +1 and go to step 3.
Step 7: return population P to main loop of the BWSA-RL algorithm

### 3.5. RL based dynamic parameter adaptation strategy

In the proposed BWSA-RL framework, reinforcement learning is employed as a supervisory control mechanism rather than as a direct solution generator. The RL component does not construct scheduling solutions or modify individual chromosomes explicitly. Instead, it dynamically regulates the evolutionary behavior of BWSA by adapting key algorithmic parameters during the optimization process.

In the proposed framework, RL is used to adaptively control two key parameters of the BWSA algorithm, namely the procreation rate $R_{p}$ and mutation rate $R_{m}$. These parameters were selected because they directly regulate the exploration–exploitation trade-off of the search process. Specifically, $R_{p}$ influences the generation of new candidate solutions and thus controls population diversity at a global level, whereas $R_{m}$ introduces stochastic perturbations that help the algorithm escape local optima. Compared to other parameters, $R_{p}$ and $R_{m}$ have the most significant impact on search dynamics while maintaining low computational overhead. Therefore, adapting these parameters through reinforcement learning enables dynamic search control without modifying the underlying solution representation or increasing algorithmic complexity. In static-parameter evolutionary algorithms, selecting suitable values for these parameters is challenging and highly problem-dependent. BWSA-RL addresses this limitation by enabling parameter adaptation based on real-time feedback from the search process.

At each generation, the RL agent observes the current search state, which reflects the population’s recent performance in terms of convergence and diversity across multiple objectives. Based on this state, the agent selects an action corresponding to a predefined range of parameter values. The selected parameters are then applied in the next evolutionary cycle. After the population is updated, a reward signal, derived from changes in hypervolume, is computed and used to update the RL policy.

To ensure stable learning and efficient convergence, a hybrid learning strategy is adopted in which SARSA is employed during the early stages of optimization, followed by Q-learning in later generations. The on-policy nature of SARSA provides conservative and stable updates when the population is highly diverse and the search landscape is uncertain. As the optimization progresses and state–action values become more reliable, Q-learning is activated to accelerate convergence through greedy exploitation of learned policies.

Through this adaptive control mechanism, reinforcement learning enables BWSA-RL to autonomously adjust its search behavior in response to problem dynamics, thereby improving robustness, scalability, and solution quality without manual parameter tuning.

#### 3.5.1. Introduction of SARSA and Q-learning.

**Fig 8:**
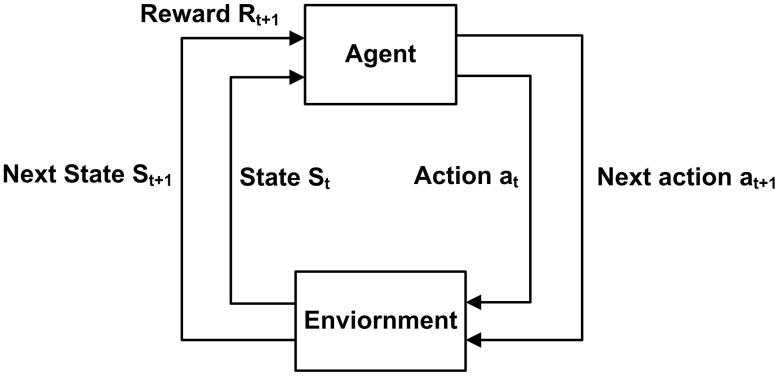
State-agent interaction diagram illustrating the reinforcement learning mechanism.

RL enables agent to learn optimal strategies by interacting with their environment. The agent observes its state, takes an action, and receives feedback as a reward. Positive rewards increase the likelihood of repeating an action, while negative rewards decrease it. Over time, the agent learns to maximize cumulative rewards for optimal decision-making. This learning action is explained in Fig 8, an agent gets the current state $S_{t}$ of the environment and takes an action $a_{t}$, due to this action the environment state changes to $S_{t+\text{1}}$ and a reward $R_{t+\text{1}}$ is returned, then based on this $S_{t+\text{1}}$ and $R_{t+\text{1}}$ the agent takes a new action $a_{t+\text{1}}$. This process goes on until the agent learns to maximize the total accumulated reward.

The framework of both SARSA and Q-learning algorithms is similar, and the only difference is in the calculation of future reward for updating of Q-tables. Equations 16 and 17 are for updating Q-tables for SARSA and Q-learning respectively.


$$Q\left(S_{t},a_{t}\right)\leftarrow Q\left(S,a_{t}\right)+\alpha \left(r_{t+\text{1}}+\gamma Q\left(S_{t+\text{1}},a_{t+\text{1}}\right)-Q\left(S_{t},a_{t}\right)\right)$$ (16)

$$Q\left(S_{t},a_{t}\right)\leftarrow Q\left(S_{t},a_{t}\right)+\alpha \left(r_{t+\text{1}}+\gamma \;\underset{a}{\text{max}}Q\left(S_{t+\text{1}},a\right)-Q\left(S_{t},a_{t}\right)\right)$$ (17)

Where $Q\left(S_{t},a_{t}\right)$ is the Q-table value for state $S_{t}$ and action $a_{t}$, α is the learning rate and γ is the discount factor used for deduction of future rewards. Q-learning, an off-policy method, updates Q-values based on maximum future rewards for faster convergence but risks local optima. SARSA, an on-policy approach, learns more cautiously, reducing the chance of local optima but converging more slowly.

#### 3.5.2. Conversion condition operator: a SARSA to Q-learning switching mechanism.

This study utilizes both SARSA and Q-learning for updating Q-tables. Initially BWSA-RL uses SARSA algorithm to update Q-tables, but after certain time the algorithm switches to Q-learning, the conversion condition of this transition is based on the proposed sparseness S, defined by the following equation;


$$S=\frac{\overset{P}{\underset{\text{0}}{Q}}+\overset{M}{\underset{\text{0}}{Q}}}{\text{2}|States||Actions|}$$ (18)

Where $\overset{P}{\underset{\text{0}}{Q}}$ and $\overset{M}{\underset{\text{0}}{Q}}$ are number of zero values in procreation and mutation Q-tables respectively, |States| and |Actions| are the total number of states and actions respectively. Initially, all elements of the Q-table matrices in BWSA-RL are zeros, resulting in S=1. As the search progresses and the algorithm continues scoring rewards, these zeros begin to diminish, causing S to decrease. The BWSA-RL switches to Q-learning when $S\leq T_{s}$, where $T_{s}$ is the sparseness threshold.

The proposed sparseness S reflects the proportion of unvisited state–action pairs. A high S value indicates that the agent has not sufficiently explored the state–action space, making SARSA more suitable due to its on-policy nature and stable learning behavior under limited information. As learning progresses, the S decreases, indicating that the agent has accumulated sufficient experience across different states. At this stage, switching to Q-learning enables more aggressive exploitation of learned policies through its off-policy update rule. Therefore, Q-table sparseness S serves as an intuitive and data-driven indicator of the exploration maturity of the learning process.

The threshold parameter $T_{s}$ determines the point at which the learning strategy transitions from SARSA to Q-learning. A lower value of $T_{s}$ would trigger early switching, potentially leading to premature exploitation, whereas a higher value may delay the transition and increase computational overhead. Thus, $T_{s}$ represents a trade-off between exploration completeness and convergence speed. The value of $T_{s}$ is empirically determined through Taguchi design of experiment in Section 4.3.

#### 3.5.3. State definition.

In context of RL, the state can be considered as agent’s modeling and encoding of its environment [36]. An ideal methodology to define state must encompass all characteristics of the population, and the complexity of defining states for multi-objective algorithms becomes more challenging [2]. This research purposes a state definition based on changes in average values of all four optimization objectives, $\Delta \overset{―}{MK}$, $\Delta \overset{―}{TEC}$, $\Delta \overset{―}{ADP}$ and $\Delta \overset{―}{INS}$ are defined as difference in average MK, TEC, ADP and INS for previous and current generations. The formal expressions for these differences are given in equation 19–22.


$$\Delta \overset{―}{MK}=\frac{\sum_{i=\text{1}}^{N}\overset{g}{\underset{i}{MK}}-\sum_{i=\text{1}}^{N}\overset{g-\text{1}}{\underset{i}{MK}}}{N}\;$$ (19)

$$\Delta \overset{―}{TEC}=\frac{\sum_{i=\text{1}}^{N}\overset{g}{\underset{i}{TEC}}-\sum_{i=\text{1}}^{N}\overset{g-\text{1}}{\underset{i}{TEC}}}{N}\;$$ (20)

$$\Delta \overset{―}{ADP}=\frac{\sum_{i=\text{1}}^{N}\overset{g}{\underset{i}{ADP}}-\sum_{i=\text{1}}^{N}\overset{g-\text{1}}{\underset{i}{ADP}}}{N}\;$$ (21)

$$\Delta \overset{―}{INS}=\frac{\sum_{i=\text{1}}^{N}\overset{g}{\underset{i}{INS}}-\sum_{i=\text{1}}^{N}\overset{g-\text{1}}{\underset{i}{INS}}}{N}\;$$ (22)

Where $\overset{g}{\underset{i}{MK}}$ and $\overset{g-\text{1}}{\underset{i}{MK}}$ represent makespan of i^th^ member of population for g^th^ and (g-1)^th^ generation respectively. $\overset{g}{\underset{i}{TEC}}$, $\overset{g-\text{1}}{\underset{i}{TEC}}$, $\overset{g}{\underset{i}{ADP}}$, $\overset{g-\text{1}}{\underset{i}{ADP}}$, $\overset{g}{\underset{i}{INS}}$ and $\overset{g-\text{1}}{\underset{i}{INS}}$ are defined similarly. N is the total number of solutions in population. Following the calculation of these delta values, Table 3 is utilized to assess the current state of the population.

**Table 3:** State definition table.

State	MK	TEC	ADP	INS
1	$\Delta \overset{―}{MK}\geq \text{0}$	$\Delta \overset{―}{TE}\geq \text{0}$	$\Delta \overset{―}{ADP}\geq \text{0}$	$\Delta \overset{―}{INS}\geq \text{0}$
2	$\Delta \overset{―}{MK}\geq \text{0}$	$\Delta \overset{―}{TE}\geq \text{0}$	$\Delta \overset{―}{ADP}\geq \text{0}$	$\Delta \overset{―}{INS}<\text{0}$
3	$\Delta \overset{―}{MK}\geq \text{0}$	$\Delta \overset{―}{TE}\geq \text{0}$	$\Delta \overset{―}{ADP}<\text{0}$	$\Delta \overset{―}{INS}\geq \text{0}$
4	$\Delta \overset{―}{MK}\geq \text{0}$	$\Delta \overset{―}{TE}\geq \text{0}$	$\Delta \overset{―}{ADP}<\text{0}$	$\Delta \overset{―}{INS}<\text{0}$
5	$\Delta \overset{―}{MK}\geq \text{0}$	$\Delta \overset{―}{TE}<\text{0}$	$\Delta \overset{―}{ADP}\geq \text{0}$	$\Delta \overset{―}{INS}\geq \text{0}$
6	$\Delta \overset{―}{MK}\geq \text{0}$	$\Delta \overset{―}{TE}<\text{0}$	$\Delta \overset{―}{ADP}\geq \text{0}$	$\Delta \overset{―}{INS}<\text{0}$
7	$\Delta \overset{―}{MK}\geq \text{0}$	$\Delta \overset{―}{TE}<\text{0}$	$\Delta \overset{―}{ADP}<\text{0}$	$\Delta \overset{―}{INS}\geq \text{0}$
8	$\Delta \overset{―}{MK}\geq \text{0}$	$\Delta \overset{―}{TE}<\text{0}$	$\Delta \overset{―}{ADP}<\text{0}$	$\Delta \overset{―}{INS}<\text{0}$
9	$\Delta \overset{―}{MK}<\text{0}$	$\Delta \overset{―}{TE}\geq \text{0}$	$\Delta \overset{―}{ADP}\geq \text{0}$	$\Delta \overset{―}{INS}\geq \text{0}$
10	$\Delta \overset{―}{MK}<\text{0}$	$\Delta \overset{―}{TE}\geq \text{0}$	$\Delta \overset{―}{ADP}\geq \text{0}$	$\Delta \overset{―}{INS}<\text{0}$
11	$\Delta \overset{―}{MK}<\text{0}$	$\Delta \overset{―}{TE}\geq \text{0}$	$\Delta \overset{―}{ADP}<\text{0}$	$\Delta \overset{―}{INS}\geq \text{0}$
12	$\Delta \overset{―}{MK}<\text{0}$	$\Delta \overset{―}{TE}\geq \text{0}$	$\Delta \overset{―}{ADP}<\text{0}$	$\Delta \overset{―}{INS}<\text{0}$
13	$\Delta \overset{―}{MK}<\text{0}$	$\Delta \overset{―}{TE}<\text{0}$	$\Delta \overset{―}{ADP}\geq \text{0}$	$\Delta \overset{―}{INS}\geq \text{0}$
14	$\Delta \overset{―}{MK}<\text{0}$	$\Delta \overset{―}{TE}<\text{0}$	$\Delta \overset{―}{ADP}\geq \text{0}$	$\Delta \overset{―}{INS}<\text{0}$
15	$\Delta \overset{―}{MK}<\text{0}$	$\Delta \overset{―}{TE}<\text{0}$	$\Delta \overset{―}{ADP}<\text{0}$	$\Delta \overset{―}{INS}\geq \text{0}$
16	$\Delta \overset{―}{MK}<\text{0}$	$\Delta \overset{―}{TE}<\text{0}$	$\Delta \overset{―}{ADP}<\text{0}$	$\Delta \overset{―}{INS}<\text{0}$

#### 3.5.4. Action set.

This study employs two action sets comprising potential values for parameters $R_{p}$ and $R_{m}$, with each action set containing eight options. The typical values of $R_{p}$ and $R_{m}$ are in the range of 0.4 to 0.8 and 0.05 to 0.45 respectively, and interval size is set at 0.05. The eight actions for parameter $R_{p}$ are [0.4, 0.45), [0.45, 0.50), [0.50, 0.55), [0.55, 0.60), [0.60, 0.65), [0.65, 0.70), [0.70, 0.75) and [0.75, 0.80]. Similarly, the eight actions for parameter $R_{m}$ are [0.05, 0.10), [0.10, 0.15), [0.15, 0.20), [0.20, 0.25), [0.25, 0.30), [0.30, 0.35), [0.35, 0.40) and [0.40, 0.45]. For example, if the second action is selected for $R_{p}$ then the value of this parameter is set randomly between 0.45 and 0.50. The action values for $R_{m}$ are calculated in a similar manner. The expressions for calculation of $R_{p}$ and $R_{m}$ are given in equations 23 and 24.


$$R_{p}={PA}_{s}+\left({PA}_{E}-{PA}_{s}\right)\times r\;$$ (23)

$$R_{m}={MA}_{s}+\left({MA}_{E}-{MA}_{s}\right)\times r\;$$ (24)

Where ${PA}_{s}$, ${PA}_{E}$, ${MA}_{s}$ and ${MA}_{E}$ are the interval start and end values of the selected procreation and mutation action respectively, and r is a random number between [0, 1].

#### 3.5.5. Reward method.

After execution of the selected action $a_{t}$, the environment generates feedback in the form of a reward $R_{t+\text{1}}$ which determines the quality of the selected action $a_{t}$, for the state $S_{t}$. This $R_{t+\text{1}}$ could be positive, indicating improvement, or it could be negative, indicating detriment in the overall health of the population. This study employs hyper-volume ratio (HVR) to calculate reward, Hyper-volume (HV) is a measure of both convergence and diversity [37]. The expressions to calculate HVR and reward $R_{t+\text{1}}$ are given in expression 25 and 26 respectively.


$$HVR=\frac{{HV}^{g}}{{HV}^{g-\text{1}}}\;$$ (25)

$$R_{t+\text{1}}=\left\{ \begin{array}{c} \text{1}\text{0}\times HVR,\;if\;HVR>\text{1}\\ \text{0},\;otherwise \end{array}\right.\;$$ (26)

Where ${HV}^{g}$ and ${HV}^{g-\text{1}}$ are the HV of $g^{th}$ and ${\left(g-\text{1}\right)}^{th}$ generation respectively, HVR is the ration of ${HV}^{g}$ and ${HV}^{g-\text{1}}$. Instead of using reward as fixed constant value, this study uses a dynamic reward based on current value of HVR, for higher values of HVR more reward is given and vice versa. Negative rewards are not used, as these rewards might cause convergence instability [38].

#### 3.5.6. Epsilon greedy policy.

The action selection policy $\pi \left(S_{t},a_{t}\right)$ governs the agent’s selection of an action $a_{t}$ in the given state $S_{t}$. This study incorporates the Epsilon greedy policy, it maintains a balance between exploration and exploitation of search space through the choice of parameter ε. The action is selected as per the following expression.


$$\pi \left(S_{t},a_{t}\right)=\left\{ \begin{array}{c} \underset{a}{\text{max}}Q\left(S_{t},a_{t}\right)\;if\;\epsilon \geq r\\ Randomly\;a\in A \end{array}\;\right.\;$$ (27)

Where ε is the greedy rate, r is a random number between range [0,1] and A is the action set. If ϵ≥r then for the current state, action with max Q-value is selected otherwise action is selected randomly.

### 3.6. Diversity preservation with Hybrid cosine distance (HCD)

Non-dominated sorting ranks solutions into Pareto fronts, with the best front assigned rank 1 and subsequent fronts ranked in ascending order. Solutions within the same rank are non-dominating and considered equally optimal. To differentiate between them, a crowding distance metric is used [39], measuring solution diversity in objective and variable spaces. A higher value of crowding distance indicates a more promising solution within the same rank. Maintaining diversity is crucial for ensuring the effectiveness and efficiency of evolutionary algorithms.

This study proposed a novel hybrid cosine distance metric (HCD). The traditional method to evaluate crowding distance is to calculate Euclidean distance between reference solution and its neighbors in objective space. This traditional method has two limitations, first it relies on Euclidean distance which some time might be misleading due to consideration of scale [40], second MODFJSP is a discrete combinatorial optimization problem therefore many different permutations in variable space may point to the same solution in objective space. The proposed HCD overcomes these limitations by utilization of cosine distance in place of Euclidean distance. This reliance on cosine distance results in better estimate, due to consideration of angle, of separation between two vectors. Additionally, this proposed HCD hybridizes cosine distances of both variable and objective domains. The expressions to calculate ${HCD}_{i}$ for $i^{th}$ member of the population is given below.


$${CD}_{OSV}=\text{1}-\frac{{\vec{OSV}}^{i}\cdot {\vec{OSV}}^{i+\text{1}}}{\left|{\vec{OSV}}^{i}\right|\left|{\vec{OSV}}^{i+\text{1}}\right|}\;$$ (28)

$${CD}_{MAV}=\text{1}-\frac{{\vec{MAV}}^{i}\cdot {\vec{MAV}}^{i+\text{1}}}{\left|{\vec{MAV}}^{i}\right|\left|{\vec{MAV}}^{i+\text{1}}\right|}$$ (29)

$${CD}_{Obj}=\text{1}-\frac{{\vec{f}}^{i}\cdot {\vec{f}}^{i+\text{1}}}{\left|{\vec{f}}^{i}\right|\left|{\vec{f}}^{i+\text{1}}\right|}$$ (30)

$${HCD}_{i}=\frac{{CD}_{OSV}+{CD}_{MAV}}{\text{2}}+{CD}_{Obj}\;$$ (31)

Where ${CD}_{OSV}$, ${CD}_{MAV}$ and ${CD}_{Obj}$ are cosine distances of operation sequence, machine assignment and objective function vectors respectively. ${\vec{OSV}}^{i}$, ${\vec{OSV}}^{i+\text{1}}$, ${\vec{MAV}}^{i}$, ${\vec{MAV}}^{i+\text{1}}$, ${\vec{f}}^{i}$ and ${\vec{f}}^{i+\text{1}}$ represents OSV, MAV and objective values vector for $i^{th}$ and ${\left(i+\text{1}\right)}^{th}$ solution respectively. The pseudocode for calculation of HCD is given in listing Algorithm 1.

**Algorithm 1:** **Hybrid Cosine Distance (HCD) Operator**

***Input*** All Pareto Fronts *Pareto Fronts*

***Output*** Crowding Distance Data Structure *HCD*

1 *HCD* ⇓ Initialize with zeros

2 *For Each PF in Pareto Fronts*

3 Sort *PF* by objective functions f1, f2, f3, f4

4 *N* ⇓ Count of total individuals in *PF*

5 *For i* = 1 to *N*

6 ${CD}_{OSV}$ = cosine distance between OSV of P PF [i] and PF [i+1]

7 ${CD}_{MAV}$ = cosine distance between MAV of P PF [i] and PF [i+1]

8 ${CD}_{Obj}$ = cosine distance between objectives of P PF [i] and PF [i+1]

9 $HCD[i]=\frac{\left({CD}_{OSV}+{CD}_{MAV}\right)}{\text{2}}+{CD}_{Obj}$

10 End For

11 End For Each

12 Return HCD

13 End

### 3.7. Dynamic event handling and rescheduling heuristic

Real-world manufacturing shops face multiple disruptions, including new job insertions, tool wear, machine breakdowns, job cancellations, priority changes, and process alterations, adding complexity to scheduling problem [41]. To align with practical scenarios, this study focuses on the most common disruption, new job insertion, in MODFJSP. Such disruptions introduce instability, defined as the deviation from the original schedule, impacting resource organization, tooling, and personnel planning. High instability leads to excessive change management, increased costs, missed due dates, and resource wastage. Therefore, an efficient rescheduling technique must prioritize minimizing instability to maintain schedule reliability and operational efficiency.

**Fig 9:**
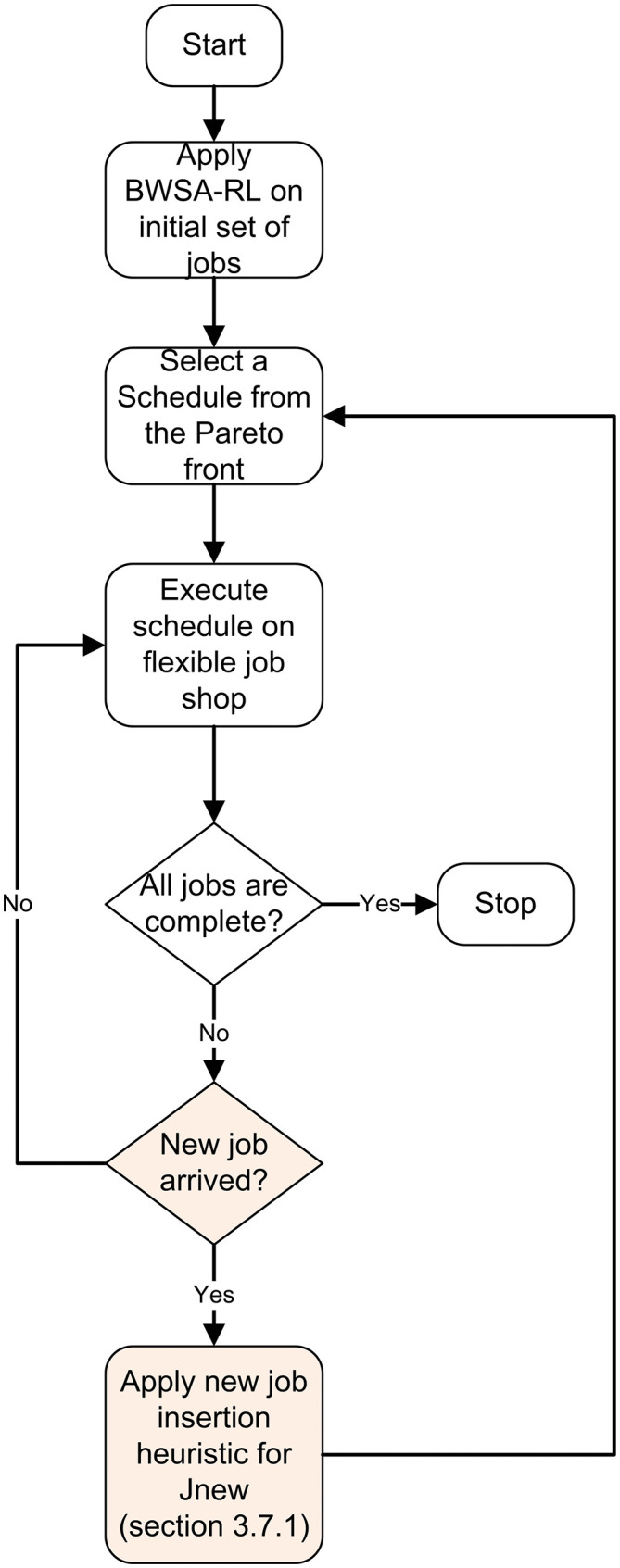
Proposed rescheduling framework for handling the insertion of new jobs in a dynamic scheduling environment.

The rescheduling framework begins with scheduling the initial job set using BWSA-RL, optimizing MK, TEC, and ADP. After execution, a Pareto front of elite solutions is generated, from which production managers select a schedule for implementation. This schedule remains active until an external disruption occurs. When a disruption arises, it is integrated into the running schedule using the proposed heuristic, ensuring minimal instability. The detailed framework is given in Fig 9.

#### 3.7.1. Insertion of new job.

The proposed heuristic for incorporation of new jobs in running schedule is based on priority of the incoming job with the aim to minimize instability. The decision matrix for rescheduling is given in Table 4, the particulars of the steps required in it are given below.

**Table 4:** Priority based decision matrix for rescheduling after arrival of new job.

Priority of Job Jnew	Freeze Action	Rescheduling Action
High	All high priority jobs	All normal and low priority jobs along with Jnew
Normal	All high and normal priority jobs	All low priority jobs along with Jnew
Low	All jobs	Only schedule Jnew

Step 1: a new job $J_{new}$ arrives.
Step 2: determine the priority of $J_{new}$.
Step 3: if priority is high then go to step 4, if priority is normal then go to step 6, if priority is low then go to step 8.
Step 4: freeze all high priority jobs to their current machine assignments and timeslot allocations.
Step 5: reschedule all normal and low priority jobs along with $J_{new}$ and then go to step 10.
Step 6: freeze all high and normal priority jobs to their current machine assignments and timeslot allocations.
Step 7: reschedule all low priority jobs along with $J_{new}$ with BWSA-RL, then go to step 10.
Step 8: freeze all the jobs to their current machine assignments and timeslot allocations.
Step 9: reschedule $J_{new}$ with BWSA-RL, then go to step 10.
Step 10: go to the main loop of the rescheduling framework.

## 4. Computational results and discussions

This section provides details of extensive computational experiments and evaluations of the proposed BWSA-RL and rescheduling heuristics. The following experimental regime has been designed to assess the performance of BWSA-RL.

1. *Taguchi design of experiments* is used to determine the optimal RL hyper parameters.
2. *MILP model* is executed in IBM CPLEX and compared with BWSA-RL for validation.
3. *Conversion condition operator* is experimentally evaluated and benchmarked against two alternative approaches.
4. *Hybrid crowding distance metric (HCD)* is benchmark against two proposed crowding distance operators.
5. *Effectiveness of the ADP objective function* is evaluated, and optimal weight levels are experimentally determined.
6. *BWSA-RL is benchmarked* against four state-of-the-art algorithms published in leading journals.
7. *Rescheduling heuristic effectiveness* under dynamic conditions is tested through experiments.

The proposed algorithm is implemented in C#.NET and executed on an Intel Core i7 processor with 8 GB RAM. The source code for BWSA-RL can be downloaded from the link https://github.com/K-Akram/BWSA-RL-Code and also included in supporting of this paper as SourceCode.Zip. Before presenting the experimental results, instance generation and performance metrics are discussed in the following sections.

### 4.1. Instance generation

The proposed algorithm incorporates job priority and allows for rescheduling to incorporate new jobs. Since no existing benchmark problems cover all these aspects, thirty problems have been generated to evaluate the algorithm’s performance [42,43]. The benchmark problems range from 25 to 500 operations, 5–50 jobs and 3–20 machines. The processing times are generated in the range of 1–10 units of time. On/Off, idling and processing energies in the ranges of 0 to 0.3, 0.2 to 0.4 and 0.5 to 1.0 units of energy respectively. Additionally, each problem includes three randomly generated dynamic new job insertion events. The proposed set of problems is named P01 to P30 and can be downloaded from https://github.com/K-Akram/Problem-Set-RL-P and also included in supporting files of this paper as ProblemSet.Zip.

### 4.2. Performance metrics

This study evaluates algorithm performance using three standard metrics: Set Coverage (C), Generational Distance (GD), and Inverse Generational Distance (IGD) [44]. Given the absence of known true Pareto fronts for the benchmark problems, the best Pareto front generated across all runs is adopted as the reference front, denoted PF*.

*Set Coverage (C):* It compares two Pareto fronts in terms of dominance, let A and B are two Pareto fronts then the percentage C(A,B) is defined as;

$$C(A,B)=\frac{\left|\{\;b\in B|\exists \;a\in A:a\;dominates\;b\}\right|}{\left|B\right|}\;$$ (32)

Where |B| denotes the total solutions in Pareto front B. This metric is computed in pairs, i.e., C(A,B) and C(B,A), Pareto front A is considered dominant only if C(A,B)>C(B,A).

*Generational Distance (GD):* GD measures the average Euclidean distance from solutions in a Pareto front PF to the closest points in the benchmark front PF*. A smaller GD value indicates better algorithm performance. It is computed as follows.

$$GD(PF,{PF}^{*})=\frac{\sqrt{\sum_{y\in PF}{min}_{x\in {PF}^{*}}{\left(dist(x,y)\right)}^{\text{2}}}}{\left|PF\right|}$$ (33)

*Inverse Generational Distance (IGD):* IGD measures the average Euclidean distance from solutions in PF* to the closest points in the PF. It is computed as follows.

$$IGD\left(PF,{PF}^{*}\right)=\frac{\sum_{x\in {PF}^{*}}{min}_{y\in PF}dist(x,y)}{\left|{PF}^{*}\right|}\;$$ (34)

A lower IGD value reflects better quality of the generated Pareto front. A zero IGD indicates that every solution in PF exists in the reference front PF*, though not necessarily vice versa. When both GD and IGD equal zero, PF and PF* are entirely identical.

*Statistical Tests:* To statistically compare the performance of multiple algorithms across multiple benchmark instances, this study employs the Friedman test, a widely used non-parametric statistical test for ranking-based comparisons in metaheuristic optimization. The Friedman test is particularly suitable for stochastic optimization algorithms, as it does not assume normality of the underlying data and is robust to outliers. However, its application is justified when performance measures are obtained from repeated independent runs and when comparisons involve the same set of problem instances across all algorithms. To assess the Friedman test’s assumptions of non-normality and non-homogeneity, the Shapiro–Wilk test is used to examine the normality of the performance data, and Levene’s test is applied to evaluate the homogeneity of variances among algorithms. Together, these statistical tests establish a rigorous and appropriate framework for comparative performance evaluation, complementing the descriptive metrics C, GD and IGD used in this study.

### 4.3. Parameter calibration through Taguchi design of experiment (DOE)

**Fig 10:**
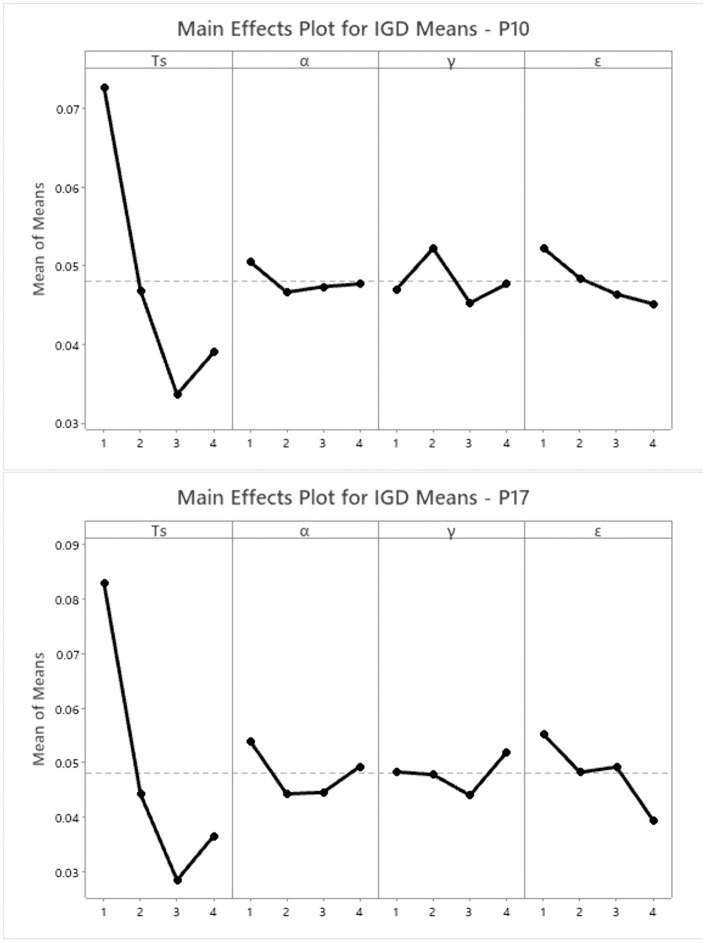
Taguchi design of experiment showing main effect plots for problems P10 and P17.

Optimizing parameter settings is crucial for maximizing algorithm performance [45]. The proposed BWSA-RL’s reinforcement learning portion has a set of four hyper parameters, i.e., sparseness threshold Ts, learning rate α, discount rate γ and epsilon greedy value ε. Four levels of each parameter are tested for two problems, P10 and P17. The levels of four parameters are listed in Table 5, and the IGD results of these tests are listed in Table 6. The analysis was conducted using Minitab (v21.2), and the main effect plots of the problems are depicted in Fig 10. Based on this analysis, the final optimal parameter values are determined as follows: Ts = 0.4, α = 0.6, γ = 0.4, and ε = 0.9.

**Table 5:** Taguchi design of experiment parameters with four selected levels.

Parameter	Levels			
	1	2	3	4
**Ts**	0.6	0.5	0.4	0.3
**α**	0.5	0.6	0.7	0.8
**γ**	0.2	0.3	0.4	0.5
**ε**	0.6	0.7	0.8	0.9

**Table 6:** IGD results for P10 and P17 for Taguchi design of experiment.

				IGD	
Ts	α	γ	ε	P10	P17
1	1	1	1	0.0779570	0.0989628
1	2	2	2	0.0789699	0.0781080
1	3	3	3	0.0601594	0.0724350
1	4	4	4	0.0736370	0.0817690
2	1	2	3	0.0563340	0.0534014
2	2	1	4	0.0340599	0.0280296
2	3	4	1	0.0530970	0.0508610
2	4	3	2	0.0437026	0.0443510
3	1	3	4	0.0336370	0.0208890
3	2	4	3	0.0298589	0.0325390
3	3	1	2	0.0368004	0.0277887
3	4	2	1	0.0342060	0.0325624
4	1	4	2	0.0340080	0.0425321
4	2	3	1	0.0435630	0.0382398
4	3	2	4	0.0391925	0.0270028
4	4	1	3	0.0391140	0.0383140

### 4.4. Validation of proposed BWSA-RL model

The proposed model’s validity was tested on ten small-scale benchmark problems (S01 to S10) with total operations ranging from 4 to 16, solved using IBM ILOG CPLEX optimization studio (v22.1.1). It is important to note that these small-scale problems are selected due to the exponential computational complexity of exact methods. Benchmark instances are accessible via the link given in Section 5.1. Due to CPLEX’s intrinsic limitation of simultaneous optimization of multiple objectives, three primary objectives, i.e., makespan MK, total energy consumption TEC, and average due-date penalty ADP were optimized individually using OPL. CPLEX was configured with a 3600 second runtime limit, after which it terminates if an optimal solution was not found.

For comparison, BWSA-RL was run with a population size of 180, a maximum of 200 generations, Ts = 0.4, α = 0.6, γ = 0.4, and ε = 0.9. Table 7 summarizes the best results achieved by each algorithm on the optimization objectives and lists the total number of Pareto front solutions produced by BWSA-RL. CPLEX solved instances S01 to S09 optimally but was unable to solve S10. Notably, BWSA-RL not only found optimal solutions for all solvable instances faster than CPLEX but also outperformed CPLEX on S10. These results suggest that while CPLEX is suitable for smaller problem instances but more sophisticated algorithms like BWSA-RL are necessary for larger-scale problems. The CPLEX results should be interpreted as a baseline stability check rather than a comprehensive validation of multi-objective performance.

**Table 7:** CPLEX versus BWSA-RL: Test results for problems S01 to S10.

Instance	Total operations	CPLEX				BWSA-RL				
		MK	TEC	ADP	Time	MK	TEC	ADP	Time	PF size
S01	4	26	184.5	0.0	0.1	26	184.5	0.0	11.09	7
S02	6	35	616.8	0.3	0.9	35	616.8	0.3	12.58	11
S03	8	56	812.6	0.2	15.0	56	812.6	0.2	15.23	13
S04	8	48	811.7	0.8	42.2	48	811.7	0.8	12.55	4
S05	10	59	606.7	1.26	180.9	59	606.7	1.26	13.34	12
S06	12	72	1321.6	0.47	269.5	72	1321.6	0.47	13.34	27
S07	12	57	846.2	0.975	424.7	57	846.2	0.975	14.42	17
S08	15	53	841.5	0.033	2481.1	53	841.5	0.033	15.97	60
S09	15	47	840.1	0.46	3357.8	47	840.1	0.46	13.3	32
S10	16	56	706.3	1.1	3600.0	54	705.1	0.25	13.95	73

### 4.5. Conversion condition operator testing and benchmarking

The proposed conversion operator, designed to switch from SARSA to Q-learning, was evaluated and benchmarked against two alternatives. Three versions of BWSA-RL were implemented for comparison: BWSA-RL (A) with the proposed conversion operator, BWSA-Fixed (B) which switches to Q-learning after completing half of the total generations, and, BWSA-SLGA (C) with the conversion operator proposed by [46], the expression for this operator is given in equation 35.


$$SLGA\;Operator=\left\{ \begin{array}{c} @cSARSA\;N_{ti}<\frac{N_{ts}\times N_{ta}}{\text{2}}\mathrm{\backslash vspace}1mm\\ Q-learning\;N_{ti}\geq \frac{N_{ts}\times N_{ta}}{\text{2}} \end{array}\;\right.\;$$ (35)

where $N_{ti}$ represents the number of current iterations. $N_{ts}$ represents the total number of states and $N_{ta}$ represents the total number of actions. To minimize the influence of random factors, each benchmark problem was executed 30 times using a population size of 180 and a maximum of 200 generations. Three performance metrics were calculated for each problem.

**Fig 11:**
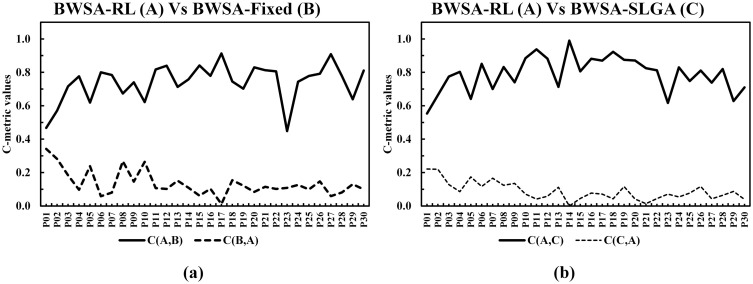
C-metric comparison of the proposed conversion operator (BWSA-RL) with BWSA-Fixed and BWSA-SLGA operators.

**Fig 12:**
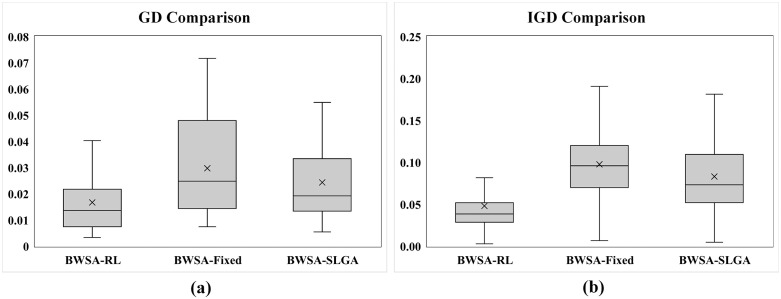
Comparison of GD and IGD metrics for the proposed conversion operator (BWSA-RL) against BWSA-Fixed and BWSA-SLGA operators.

The C-metric data comparison of proposed conversion operator with fixed and SLGA operators are presented in Fig 11a and Fig 11b respectively. The data shows that the proposed operator completely outperformed the other two benchmarking approaches. Comparison of GD and IGD is shown in Fig 12, lower GD values suggest that BWSA-RL achieves closer proximity to the true Pareto front compared to BWSA-Fixed and BWSA-SLGA. Similarly, the lower IGD values for BWSA-RL indicate better distribution and diversity of solutions along the Pareto front. The performance metrics highlight the superior convergence and spread of BWSA-RL in comparison to the fixed and SLGA operators. The complete data of all three metrics are given in S1 Table.

The Friedman test was employed to statistically evaluate the performance differences among the compared algorithms. Prior to its application, the underlying assumptions regarding data distribution were examined. Specifically, Levene’s test was conducted to assess the homogeneity of variances, while the Shapiro–Wilk test was used to examine data normality. As reported in Table 8, Levene’s test yielded a p-value less than 0.05, indicating significant variance heterogeneity among the datasets, and the Shapiro–Wilk test results confirmed that the IGD data deviate from normality. These findings justify the adoption of a non-parametric statistical approach. Consequently, the Friedman test was applied to the IGD results, and the obtained p-value (p < 0.05) indicates that the observed performance differences among the algorithms are statistically significant and not attributable to random variation. Furthermore, the mean rank values presented in Table 9 demonstrate that the proposed BWSA-RL method achieves the best overall ranking, confirming its superior performance compared to the other benchmarking conversion condition operators.

**Table 8:** Levene and Shapiro-Wilk test results for IGD data of comparison with other conversion condition operators.

Levene Test for non-homogeneity of datasets						
Source of Variation	Sum of Squares	Degrees of freedom	Mean Sum	F	P-value	F critical
Between Groups	0.03909	2	0.01955	13.55929	0.00001	3.10130
Within Groups	0.12542	87	0.00144			
**Shapiro-Wilk Test for non-normality of datasets**						
**Algorithm**	**BWSA-RL**	**BWSA-Fixed**	**BWSA-SLGA**			
W Statistic	0.799	0.941	0.974			
p-value	0.009	0.010	0.011			

**Table 9:** Friedman test results for IGD values of comparison with other conversion condition operators.

Algorithm	Mean rank value	Priority
BWSA-Fixed	2.83	3
BWSA-SLGA	2.17	2
BWSA-RL	1.0	1
Test Statistics	51.7	
N	30	
Chi-Square	5.99	
Degrees of freedom	3	
p-value	0.000	

The superior performance of BWSA-RL can be attributed to the proposed dynamic sparsity-based operator, which adjusts the switching process from SARSA to Q-learning based on the learning itself. Unlike the fixed operator, which prematurely transitions to Q-learning halfway through the generations, or the SLGA operator, which follows a static approach, the dynamic operator ensures adaptability and balances exploration and exploitation effectively. In conclusion, the results validate the efficacy of the proposed conversion operator.

### 4.6. Effectiveness of hybrid crowding distance metric (HCD)

The HCD is benchmarked against two other crowding distance metrics proposed in well reputed journals. These selected benchmarking crowding distance metrics are modified crowding distance operator (MCDO) proposed by [6] and Hamming distance and Euclidean distance (HDED) proposed by [8]. Three versions of BWSA-RL were coded using HCD, MCDO and HDED, and these versions were designated as HCD (A), MCDO (B), and HDED (C). To eliminate any chance factor each benchmark instance was run 30 times and performance metrics were calculated for each run.

**Fig 13:**
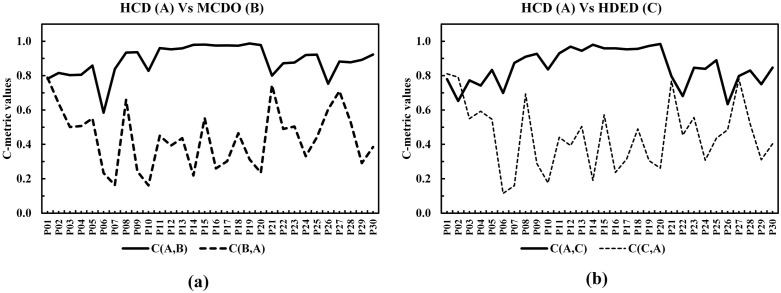
C-metric comparison between (a) HCD and MCDO and (b) HCD and HDED, highlighting the superior performance of HCD.

**Fig 14:**
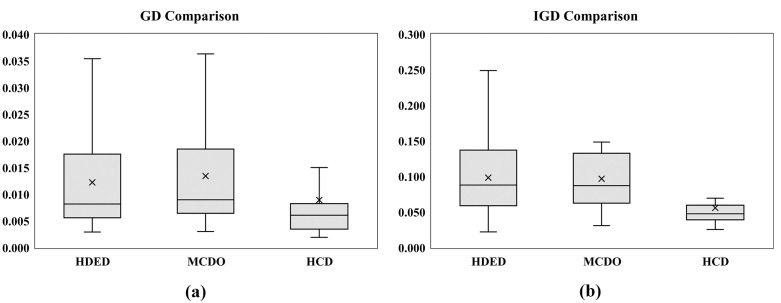
GD and IGD comparison of HCD with MCDO and HDED using (a) box plots of GD and (b) box plots of IGD metrics.

The C-metric values, presented in graphical format in Fig 13, clearly demonstrate that the results obtained using the HCD approach are superior to those achieved with other benchmark crowding distance metrics. HDED performed better for problems P01 and P02 but as the problem size increased HCD started producing better results. The GD and IGD values for the three methods are summarized in box plots shown in Fig 14. These visual representations provide a clear comparison, highlighting the performance advantages of the proposed HCD approach. Specifically, HCD consistently achieves lower GD and IGD values compared to HDED and MCDO, emphasizing its ability to generate Pareto fronts with enhanced convergence and diversity. Moreover, the tighter spread of HCD results, as reflected in the compact interquartile range, showcases its robustness and stability across different runs. These findings validate the effectiveness of the HCD method in producing high-quality, well-distributed solutions. The complete data of all three metrics are given in S2 Table.

To examine whether statistically significant differences exist among the evaluated crowding distance metrics, a Friedman non-parametric test was performed. Before conducting this analysis, the distributional characteristics of the IGD data were investigated. Variance homogeneity was assessed using Levene’s test, while the normality of the data was evaluated using the Shapiro–Wilk test. As summarized in Table 10, Levene’s test indicates significant variance heterogeneity (p < 0.05), and the Shapiro–Wilk test shows deviations from normality for most metrics. Although the non-normality assumption is violated for the HDED metric (p > 0.05), this isolated case does not invalidate the use of the Friedman test. These observations collectively support the selection of a non-parametric statistical framework. The Friedman test results, shown in Table 11, yielded a p-value less than 0.05, confirming that the observed performance differences among the crowding distance metrics are statistically significant. Moreover, the mean rank analysis reveals that the proposed HCD consistently attains the best ranking, indicating its superior performance relative to the other benchmark crowding distance measures.

**Table 10:** Levene and Shapiro-Wilk test results for IGD data of crowding distance comparison.

Levene Test for non-homogeneity of datasets						
Source of Variation	Sum of Squares	Degrees of freedom	Mean Sum	F	P-value	F critical
Between Groups	0.006471	2	0.003235	3.72194	0.028121	3.101296
Within Groups	0.075624	87	0.000869			
**Shapiro-Wilk Test for non-normality of datasets**						
Algorithm	**HCD**	**MCDO**	**HDED**			
W Statistic	0.607	0.910	0.940			
p-value	0.007	0.016	0.111			

**Table 11:** Friedman test results for IGD data of crowding distance comparison.

Algorithm	Mean rank value	Priority
MCDO	2.6	3
HDED	2.4	2
HCD	1.0	1
Test Statistics	42.5	
Number of samples	30	
Chi-Square	6.0	
Degrees of freedom	2	
p-value	0.000	

The results highlight the effectiveness and utility of the HCD approach. This superior performance is primarily attributed to its method of assessing solution scarcity around the reference point, which effectively integrates information from both the variable and objective domains. Additionally, the use of cosine distance further improves the results by addressing scaling issues, a common limitation of the Euclidean distance.

### 4.7. Effectiveness of weights on due date compliance

This section gauges the effect of various weight settings on average due-date penalty (ADP) function, stated in equation 3. The ADP functions have three control weights $w_{H}$, $w_{N}$ and $w_{L}$ for high, normal and low priority jobs. To test the effects of these weights on non-compliance (NC) of due dates, MK, TEC and ADP following test has been designed.

Step 1: due date $D_{i}$ for each job $J_{i}$ is calculated with the following equation.


$$D_{i}=R_{f}\sum_{j=\text{1}}^{n_{i}}\left(\frac{\sum_{k=\text{1}}^{m}t_{i,j,k}W_{i,j,k}}{\sum_{k=\text{1}}^{m}W_{i,j,k}}\right)\;$$ (36)

Where $R_{f}$ is the relaxation factor, for this study its value is assumed to be 1.2, $n_{i}$ is the total number of operations for $i^{th}$ job, m is the total number of machines, $t_{i,j,k}$ is time for operation $O_{i,j}$ on machine k and $W_{i,j,k}=\text{1}$ if $O_{i,j}$ is performed on machine k otherwise $W_{i,j,k}=\text{0}$.

Step 2: five setting levels of weights are selected such that $w_{H}>w_{N}>w_{L}$ and $w_{H}+w_{N}+w_{L}=\text{1}$. These weights are listed in Table 12.

**Table 12:** Setting of weights to evaluate effectiveness ADP objective on due-date compliance.

Setting	W_H_	W_N_	W_L_
S1	0.33	0.33	0.33
S2	0.5	0.3	0.2
S3	0.5	0.4	0.1
S4	0.6	0.3	0.1
S5	0.7	0.2	0.1

**Fig 15:**
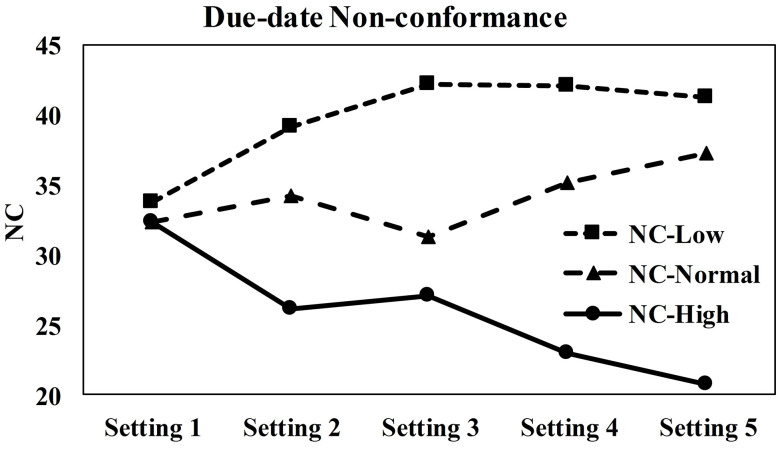
Comparison of average due-date non-conformance across different weight configurations.


Step 3: all benchmark problems are solved 30 times with each weight setting and NC of due dates NC_H_, NC_N_ and NC_L_ for high, normal and low priority jobs are calculated through equations 37–39. The average NC values for each weight setting are shown in Fig 15.

$${NC}_{H}=\frac{\text{1}}{\left|PF\right|}\sum_{l=\text{1}}^{\left|PF\right|}\left(\frac{\sum_{i=\text{1}}^{N}\left\{ \begin{array}{c} @l\left|D_{i}-C_{l,i}\right|,\;if\;priority\;of\;J_{i}\;is\;High\\ \text{0},\;otherwise\; \end{array}\right.\;}{\sum_{i=\text{1}}^{N}\left\{ \begin{array}{c} @l\text{1},if\;priority\;of\;J_{i}\;is\;High\;\\ \text{0},\;otherwise\; \end{array}\right.\;}\right)\;\mathrm{\backslash ]}$$ (37)

$${NC}_{N}=\frac{\text{1}}{\left|PF\right|}\sum_{l=\text{1}}^{\left|PF\right|}\left(\frac{\sum_{i=\text{1}}^{N}\left\{ \begin{array}{c} @l\left|D_{i}-C_{l,i}\right|,\;if\;priority\;of\;J_{i}\;is\;Normal\\ \text{0},\;otherwise\; \end{array}\right.\;}{\sum_{i=\text{1}}^{N}\left\{ \begin{array}{c} @l\text{1},if\;priority\;of\;J_{i}\;is\;Normal\;\\ \text{0},\;otherwise\; \end{array}\right.\;}\right)\;\mathrm{\backslash ]}$$ (38)

$${NC}_{L}=\frac{\text{1}}{\left|PF\right|}\sum_{l=\text{1}}^{\left|PF\right|}\left(\frac{\sum_{i=\text{1}}^{N}\left\{ \begin{array}{c} @l\left|D_{i}-C_{l,i}\right|,\;if\;priority\;of\;J_{i}\;is\;Low\\ \text{0},\;otherwise\; \end{array}\right.\;}{\sum_{i=\text{1}}^{N}\left\{ \begin{array}{c} @l\text{1},if\;priority\;of\;J_{i}\;is\;Low\;\\ \text{0},\;otherwise\; \end{array}\right.\;}\right)\;\mathrm{\backslash ]}$$ (39)

Where PF is the Pareto front of elite solutions, |PF| is the number of solutions in PF, N is the total number of jobs, $D_{i}$ and $C_{l,i}$ are the due-date and completion time of $i^{th}$ job for $l^{th}$ solution in PF respectively.

**Fig 16:**
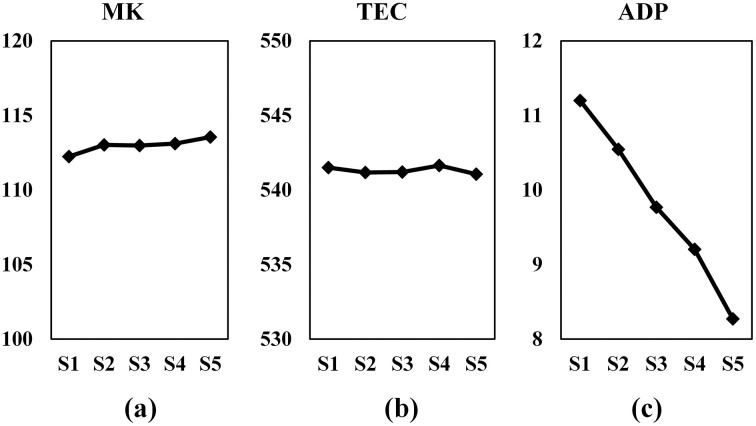
Effect of different weight settings on optimization objectives:(a) makespan, (b) total energy consumption, and (c) average due-date penalty.

Step 4: by using results obtained in this run, average MK, TEC and ADP are also calculated and results are shown in Fig 16.

By studying Fig 15 it can be observed that Setting 1 is experiment-control, and for this setting there is no significant difference in NC for all priorities, but as the weight $w_{H}$ increases the compliance of due date for high priority jobs increases.

To study the effect of weights on objective values the average MK, TEC and ADP are plotted in Fig 16. From figure, it can be inferred that the weight settings have no significant impact on MK and TEC. The ADP is affected by weight settings and the most significant parameter to effect ADP value is the weight of $w_{H}$ parameter. The complete data table for effects of weights on NC are given in S3 Table, and for effects of weights on optimization objectives are given in S4 Table.

The experimental analysis demonstrates that the weighting structure embedded in the ADP objective plays a decisive role in controlling due-date compliance, particularly for high-priority jobs. As the weight $w_{H}$ increases, the optimization process places greater emphasis on reducing deviations from due dates for urgent jobs, thereby guiding the search toward schedules that prioritize timely completion of high-priority tasks. This targeted emphasis explains the observed improvement in due-date compliance without introducing notable trade-offs in makespan or total energy consumption. Since MK and TEC are governed primarily by processing sequences and machine assignments, adjustments in due-date penalty weights do not significantly alter their behavior. The results indicate that the weighted ADP formulation provides an effective and flexible mechanism for incorporating job priorities into the scheduling process. Among the tested configurations, the weight setting $w_{H}=\text{0}.\text{5}$, $w_{N}=\text{0}.\text{4}$, and $w_{L}=\text{0}.\text{1}$ offers a balanced compromise, achieving improved compliance for urgent and normal jobs while preserving overall solution quality, making it well suited for practical multi-priority scheduling environments.

### 4.8. Comparison of BWSA-RL with other algorithms

BWSA-RL was compared with four state-of-the-art algorithms published in reputed journals. The selection criteria for benchmark algorithms were: multi-objective optimizations algorithm, preferably proposed to solve FJSP, published in high impact factor journals, and preferably a reinforcement learning based algorithm. As per the above-mentioned criteria, four algorithms were selected: 1) Evolutionary algorithm incorporating reinforcement learning (EARL), published in 2024 [2]. 2) Multi-objective Q-learning based hyper heuristic with Bi-criteria selection (QHH-BS), published in 2022 [47]. 3) Reinforcement learning multi-objective evolutionary algorithm (RMOEAD), published in 2022 [38]. 4) Enhanced non-dominated sorting genetic algorithm (ENSGA), published in 2023 [12].

**Fig 17:**
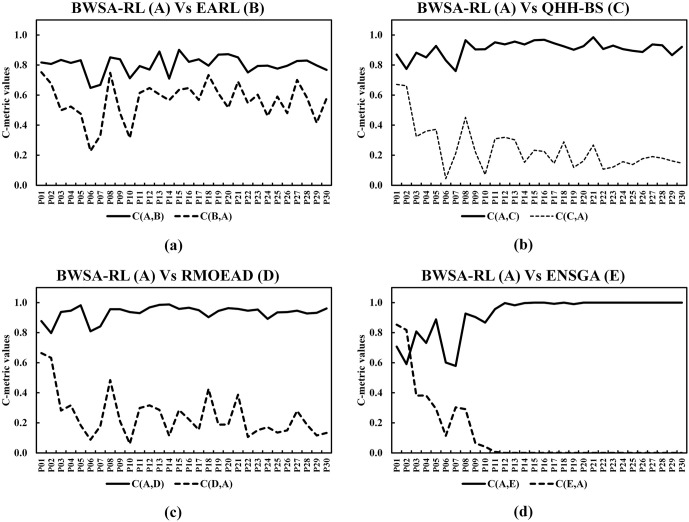
C-metric comparison of BWSA-RL with four competing algorithms:(a) EARL, (b) QHH-BS, (c) RMOEAD, and (d) ENSGA.

**Fig 18:**
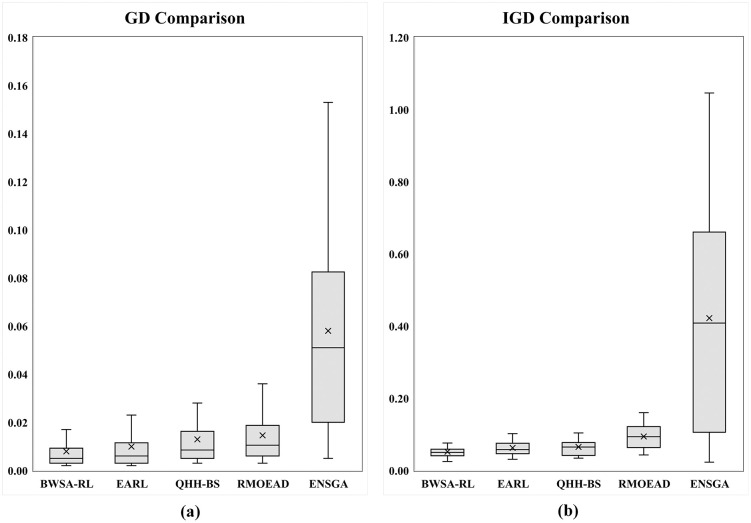
Comparison of GD and IGD metrics between BWSA-RL and competing algorithms using (a) GD box plots and (b) IGD box plots.

Algorithms [1,2], and [3] are RL-based algorithms similar to the proposed BWSA-RL, while algorithm [4] is a non-RL technique selected specifically for benchmarking against an efficient non-RL-based method. All benchmarking algorithms, except QHH-BS, were originally proposed for solving multi-objective flexible job shop scheduling problem and were implemented using the parameter configurations recommended in their respective original studies. Although QHH-BS was introduced for a closely related mixed shop scheduling environment, its underlying optimization mechanism is generic and applicable to the problem considered in this work. Each benchmarking instance was run 30 times to eliminate all chance factors, three performance metrics were calculated and shown in Table 13. The first portion of the Table 13 and Fig 17 shows all C-metric comparisons of four benchmark algorithms with BWSA-RL, from this data it is evident that BWSA-RL generated superior values of C-metric than other benchmarking algorithms, While ENSGA yielded superior C-metric results for the initial two problems, BWSA-RL demonstrated dominant performance as the problem size increased, surpassing all competing algorithms. The second and third section of Table 13 and box plot of Fig 18 compares the GD and IGD results respectively, the experimental data indicates that BWSA-RL performed consistently better than other benchmark algorithms and produced lower values of GD and IGD metrics. In the last column of Table 13, win status of BWSA-RL is listed, a win and loss are indicated by a ‘+’ and ‘-’ signs respectively. The criterion for winning is that BWSA-RL must perform better in terms of all performance metrics. As per this criterion BWSA-RL scored 25 wins out of 30 benchmark problems and the overall win rate is 83.3%. The five scored losses occurred for problems P01, P02, P04, P07 and P11, which are small-scale instances. These exceptions can be attributed to the small-scale nature of the affected instances, which limits the learning horizon available to the reinforcement learning component. Under such conditions, the adaptive policy may not fully learn before the maximum allowable iterations runout. In contrast, larger problem instances provide richer state–action interactions, enabling more effective learning of procreation and mutation strategies and resulting in superior performance.

**Table 13:** Comparison of BWSA-RL with other algorithms for performance metrics.

Problem	Set Convergence								Generational Distance					Inverse Generational Distance					
	Ratio								Mean (Standard Deviation)					Mean (Standard Deviation)						
	(A,B)	(B,A)	(A,C)	(C,A)	(A,D)	(D,A)	(A,E)	(E,A)	BWSA-RL	EARL	QHH-BS	RMOEAD	ENSGA	BWSA-RL	EARL	QHH-BS	RMOEAD	ENSGA			
P01	0.817	0.752	0.870	0.671	0.877	0.664	0.707	0.853	0.007(0.002)	0.006(0.002)	0.008(0.002)	0.009(0.002)	0.005(0.001)	0.031(0.008)	0.035(0.007)	0.034(0.007)	0.048(0.009)	0.023(0.005)	**-**
P02	0.807	0.676	0.774	0.662	0.797	0.632	0.591	0.818	0.014(0.003)	0.015(0.003)	0.016(0.002)	0.018(0.005)	0.014(0.004)	0.042(0.007)	0.047(0.008)	0.045(0.006)	0.050(0.007)	0.032(0.007)	**-**
P03	0.834	0.499	0.882	0.324	0.938	0.282	0.808	0.381	0.005(0.001)	0.007(0.003)	0.008(0.002)	0.010(0.005)	0.006(0.002)	0.056(0.009)	0.072(0.008)	0.075(0.012)	0.084(0.017)	0.078(0.025)	**+**
P04	0.814	0.524	0.851	0.360	0.946	0.316	0.732	0.382	0.007(0.002)	0.008(0.002)	0.009(0.002)	0.011(0.003)	0.007(0.002)	0.064(0.008)	0.080(0.009)	0.078(0.010)	0.085(0.009)	0.069(0.014)	**-**
P05	0.832	0.476	0.927	0.372	0.982	0.183	0.888	0.293	0.017(0.010)	0.023(0.007)	0.028(0.010)	0.032(0.010)	0.025(0.008)	0.068(0.017)	0.102(0.022)	0.104(0.019)	0.128(0.027)	0.104(0.030)	**+**
P06	0.648	0.228	0.831	0.044	0.809	0.088	0.601	0.114	0.027(0.022)	0.039(0.039)	0.052(0.029)	0.045(0.024)	0.053(0.029)	0.142(0.042)	0.157(0.039)	0.164(0.042)	0.152(0.046)	0.173(0.043)	**+**
P07	0.668	0.331	0.760	0.211	0.842	0.178	0.579	0.303	0.024(0.016)	0.037(0.023)	0.035(0.016)	0.036(0.021)	0.022(0.007)	0.076(0.014)	0.085(0.014)	0.088(0.012)	0.083(0.013)	0.080(0.022)	**-**
P08	0.851	0.748	0.965	0.452	0.956	0.484	0.927	0.291	0.006(0.001)	0.007(0.001)	0.009(0.001)	0.012(0.001)	0.012(0.004)	0.064(0.009)	0.075(0.008)	0.077(0.008)	0.121(0.019)	0.106(0.018	**+**
P09	0.838	0.480	0.904	0.222	0.956	0.210	0.904	0.064	0.006(0.002)	0.009(0.005)	0.010(0.0020	0.012(0.003)	0.014(0.004)	0.045(0.006)	0.054(0.008)	0.065(0.010)	0.066(0.006)	0.105(0.017)	**+**
P10	0.712	0.315	0.905	0.071	0.937	0.060	0.867	0.041	0.030(0.011)	0.031(0.011)	0.048(0.024)	0.049(0.024)	0.031(0.010)	0.041(0.012)	0.057(0.015)	0.091(0.024)	0.082(0.022)	0.163(0.038)	**+**
P11	0.793	0.615	0.951	0.309	0.930	0.299	0.957	0.007	0.003(0.001)	0.003(0.001)	0.005(0.001)	0.007(0.001)	0.011(0.003)	0.049(0.008)	0.053(0.008)	0.055(0.007)	0.096(0.016)	0.123(0.023)	**-**
P12	0.770	0.647	0.938	0.319	0.968	0.316	0.997	0.000	0.010(0.004)	0.011(0.004)	0.017(0.008)	0.021(0.005)	0.052(0.016)	0.059(0.010)	0.078(0.016)	0.084(0.014)	0.118(0.022)	0.293(0.077)	**+**
P13	0.890	0.604	0.956	0.302	0.985	0.286	0.982	0.000	0.006(0.001)	0.008(0.002)	0.010(0.001)	0.012(0.002)	0.041(0.008)	0.055(0.006)	0.077(0.010)	0.074(0.008)	0.105(0.014)	0.299(0.044)	**+**
P14	0.710	0.566	0.937	0.153	0.988	0.114	0.997	0.000	0.012(0.005)	0.013(0.006)	0.017(0.005)	0.023(0.005)	0.076(0.014)	0.052(0.010)	0.059(0.014)	0.060(0.010)	0.091(0.019)	0.438(0.068)	**+**
P15	0.901	0.635	0.965	0.233	0.958	0.285	1.000	0.000	0.005(0.001)	0.006(0.001)	0.009(0.006)	0.011(0.002)	0.042(0.011)	0.063(0.013)	0.080(0.012)	0.088(0.011)	0.160(0.031)	0.374(0.062)	**+**
P16	0.821	0.647	0.968	0.225	0.966	0.223	1.000	0.000	0.008(0.002)	0.009(0.002)	0.011(0.008)	0.015(0.001)	0.071(0.015)	0.059(0.014)	0.064(0.013)	0.068(0.012)	0.123(0.021)	0.440(0.058)	**+**
P17	0.838	0.568	0.945	0.146	0.950	0.154	0.992	0.001	0.004(0.001)	0.006(0.002)	0.008(0.002)	0.010(0.001)	0.044(0.010)	0.047(0.008)	0.063(0.011)	0.065(0.010)	0.102(0.016)	0.394(0.050)	**+**
P18	0.796	0.733	0.924	0.289	0.903	0.426	1.000	0.000	0.003(0.001)	0.004(0.001)	0.005(0.001)	0.006(0.001)	0.046(0.010)	0.051(0.010)	0.060(0.013)	0.065(0.010)	0.135(0.025)	0.423(0.052)	**+**
P19	0.869	0.611	0.902	0.119	0.945	0.188	0.990	0.000	0.004(0.001)	0.005(0.001)	0.006(0.001)	0.008(0.001)	0.054(0.011)	0.056(0.009)	0.074(0.011)	0.063(0.011)	0.135(0.019)	0.446(0.062)	**+**
P20	0.872	0.516	0.926	0.162	0.963	0.189	1.000	0.000	0.009(0.005)	0.016(0.007)	0.025(0.005)	0.031(0.008)	0.113(0.022)	0.043(0.008)	0.069(0.012)	0.076(0.008)	0.106(0.016)	0.680(0.084)	**+**
P21	0.851	0.690	0.985	0.268	0.958	0.387	1.000	0.000	0.004(0.001)	0.005(0.001)	0.007(0.001)	0.009(0.001)	0.102(0.023)	0.053(0.009)	0.057(0.012)	0.065(0.011)	0.153(0.025)	0.622(0.067)	**+**
P22	0.751	0.547	0.907	0.107	0.946	0.106	1.000	0.000	0.002(0.000)	0.003(0.001)	0.003(0.000)	0.003(0.000)	0.066(0.009)	0.029(0.004)	0.034(0.005)	0.036(0.003)	0.055(0.007)	0.606(0.042)	**+**
P23	0.794	0.603	0.929	0.121	0.954	0.151	1.000	0.000	0.005(0.001)	0.006(0.001)	0.007(0.001)	0.008(0.001)	0.138(0.024)	0.040(0.009)	0.045(0.0090	0.042(0.004)	0.059(0.009)	0.885(0.060)	**+**
P24	0.796	0.461	0.906	0.157	0.892	0.171	1.000	0.000	0.003(0.001)	0.005(0.000)	0.004(0.000)	0.006(0.001)	0.067(0.013)	0.047(0.010)	0.051(0.010)	0.058(0.006)	0.105(0.016)	0.594(0.032)	**+**
P25	0.776	0.590	0.895	0.138	0.935	0.135	1.000	0.000	0.003(0.001)	0.004(0.001)	0.004(0.001)	0.005(0.001)	0.116(0.027)	0.051(0.010)	0.056(0.012)	0.054(0.005)	0.071(0.012)	0.805(0.055)	**+**
P26	0.795	0.480	0.887	0.177	0.937	0.150	1.000	0.000	0.002(0.000)	0.003(0.000)	0.003(0.000)	0.004(0.001)	0.053(0.008)	0.025(0.003)	0.031(0.005)	0.038(0.004)	0.044(0.005)	0.684(0.034)	**+**
P27	0.827	0.701	0.937	0.191	0.946	0.280	1.000	0.000	0.004(0.002)	0.006(0.003)	0.011(0.003)	0.011(0.003)	0.153(0.025)	0.038(0.008)	0.041(0.009)	0.042(0.009)	0.096(0.021)	0.995(0.080)	**+**
P28	0.830	0.583	0.931	0.180	0.928	0.187	1.000	0.000	0.002(0.000)	0.003(0.000)	0.003(0.000)	0.004(0.001)	0.114(0.020)	0.043(0.008)	0.049(0.009)	0.045(0.004)	0.065(0.011)	0.929(0.068)	**+**
P29	0.797	0.415	0.866	0.162	0.932	0.116	1.000	0.000	0.002(0.000)	0.004(0.000)	0.003(0.000)	0.004(0.000)	0.050(0.010)	0.034(0.007)	0.041(0.006)	0.041(0.004)	0.060(0.006)	0.654(0.024)	**+**
P30	0.768	0.578	0.921	0.147	0.961	0.133	1.000	0.000	0.004(0.001)	0.005(0.001)	0.006(0.001)	0.006(0.001)	0.143(0.036)	0.031(0.004)	0.035(0.004)	0.036(0.004)	0.043(0.005)	1.046(0.061)	**+**
**Total Wins 83.3%**																			

**Fig 19:**
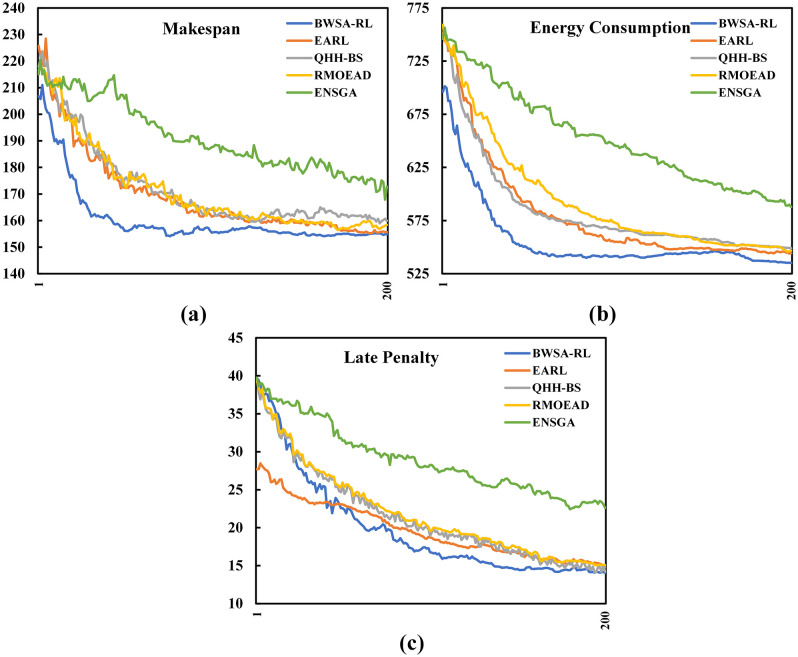
Convergence behavior of BWSA-RL for benchmark problem P15 in terms of (a) makespan, (b) total energy consumption, and (c) average due-date penalty.

To get a statistical comparison of BWSA-RL with four other algorithms, the Friedman test was performed on IGD values of compared algorithms. The non-homogeneity and non-normality were tested with Levene and Shapiro–Wilk tests, respectively, and the results were summarized in Table 14. The Levene test with a p-value < 0.05 suggested rejection of the null hypothesis, indicating that the IGD values were non-homogeneous. Similarly, the null hypothesis could also be rejected for the Shapiro–Wilk test with p-values < 0.05 for all algorithms, except RMOEAD. After establishing non-homogeneity and non-normality, the Friedman test was conducted, and the results were shown in Table 15. BWSA-RL had the highest mean rank value of 1.2 with a p-value < 0.05, indicating a significant difference in the compared algorithms’ performance. The next runners-up were EARL, QHH-BS, RMOEAD, and ENSGA with mean rank values of 2.5, 2.8, 4.1, and 4.4, respectively. Overall, the statistical results confirmed superior performance of the BWSA-RL algorithm. Fig 19 displays the convergence plots of MK, TEC, and ADP for problem P15. Visual inspection of the figure confirms that BWSA-RL converges faster to superior objective values within the same number of iterations compared to other algorithms.

**Table 14:** Levene and Shapiro-Wilk test results for IGD data of comparison with other algorithms.

Levene Test for non-homogeneity of datasets										
Source of Variation	Sum of Squares	Degrees of freedom		Mean Sum		F		P-value		F critical
Between Groups	0.003767	3		0.001256		3.967551		0.009843		2.682809
Within Groups	0.03671	116		0.000316						
**Shapiro-Wilk Test for non-normality of datasets**										
Algorithm	**BWSA-RL**		**EARL**		**QHH-BS**		**RMOEAD**		**ENSGA**	
W Statistic	0.761		0.852		0.857		0.954		0.918	
p-value	0.008		0.009		0.010		0.319		0.031	

**Table 15:** Friedman test results for IGD values of comparison with other algorithms.

Algorithm	Mean rank value	Priority
ENSGA	4.4	5
RMOEAD	4.1	4
QHH-BS	2.8	3
EARL	2.5	2
BWSA-RL	1.2	1
Test Statistics	81.2	
N	30	
Chi-Square	9.48	
Degrees of freedom	4	
p-value	0.000	

The performance of the proposed BWSA-RL is assessed using C-metric, GD, IGD, and convergence rate, and benchmarked against four state-of-the-art algorithms: EARL, QHH-BS, RMOEAD, and ENSGA. The complete examination of the data shows superior performance of BWSA-RL in solving MODFJSP, the plausible reasons for this overall gain in performance is due to multilayer novelties introduced through incorporation of reinforcement learning based parameter control, better evolutionary strategies imitating the mating behavior of black widow spiders, additionally the amalgamation of better hybrid cosine crowding distance metric have helped to boast diversity among populations. In the next section, experimental analysis of rescheduling module is discussed.

### 4.9. Effectiveness of rescheduling heuristics

**Fig 20:**
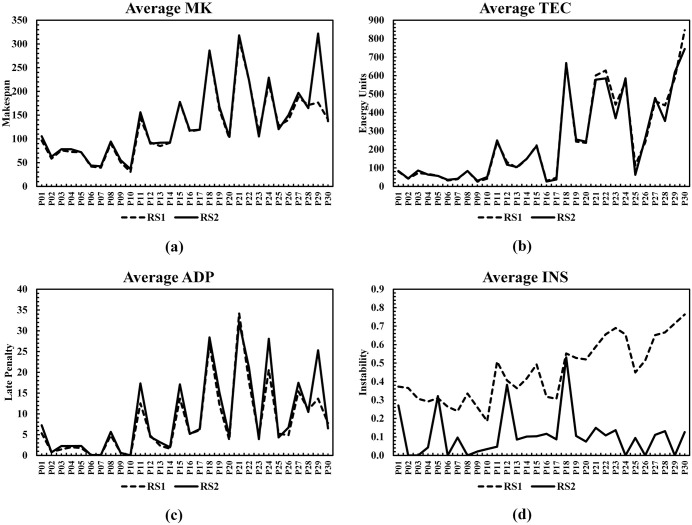
Comparative performance of complete rescheduling (RS1) and the proposed heuristic (RS2) for (a) makespan, (b) total energy consumption, (c) average due-date penalty, and (d) instability, demonstrating the superior stability of RS2 with minimal impact on other objectives.

This study of MODFJSP proposes heuristics for handling insertion of new jobs with the aim of minimizing instability objective. The proposed heuristic, referred to as RS2, was compared with the complete rescheduling technique named RS1, where all jobs were rescheduled after every disruptive event. To test the effectiveness of the rescheduling heuristic, three random job insertions were generated for each benchmark problem. Each problem was run 30 times using both RS1 and RS2 techniques to eliminate chance factors. MK, TEC, ADP, and INS were recorded for each run, with average values listed in S5 Table and data plots shown in Fig 20. Fig 20a, Fig 20b and Fig 20c show that RS1 and RS2 produced comparable results in terms of MK, TEC, and ADP, with RS1 having a slight edge. Fig 20d demonstrates that RS2 significantly outperforms RS1 in maintaining schedule stability, and as the instance size grows the performance of RS2 becomes more significant. From the data analysis, it can be inferred that the differences in MK, TEC, and ADP are minimal, but the schedules produced by RS2 are significantly more robust in terms of stability.

To statistically assess the differences between RS1 and the proposed heuristic RS2, variance-based significance testing was conducted for all optimization objectives. The p-values reported in Table 16 indicate that the differences observed for MK, TEC, and ADP are not statistically significant, as all corresponding p-values exceed the 0.05 significance level. This suggests that the performance of RS2 remains comparable to RS1 with respect to these objectives. In contrast, the p-value associated with INS < 0.05, indicating a statistically significant difference between the two rescheduling strategies. This result confirms that RS2 achieves a significant improvement in schedule stability compared to complete rescheduling. A more detailed breakdown of the statistical outcomes for benchmark instances is provided in the online supplementary material S5 Table.

**Table 16:** P-value comparison of variance for the results of RS1 and RS2 for all optimization objectives.

	MK	TEC	ADP	INS
P-value	0.690902	0.88927	0.571476	0.000000

The proposed rescheduling heuristics effectively maintain system stability without significantly affecting MK, TEC, and ADP. This is achieved through a freeze-and-reschedule strategy, which preserves optimized operations while only rescheduling necessary ones. By maintaining prior optimization benefits, the approach ensures minimal disruption and allows further optimization for new and lower-priority jobs. This results in robust and efficient performance in dynamic scheduling scenarios.

### 4.10. Managerial insights

This study presents BWSA-RL, an RL-based metaheuristic designed for MODFJSP, featuring dynamic adaptation of procreation and mutation rates, an improved hybrid crowding distance operator, and a heuristic for managing new job insertions. The algorithm autonomously adjusts parameters based on population dynamics, enhancing search efficiency. Job priority integration minimizes earliness and tardiness, making it adaptable for diverse priority levels. Tested on small to large-scale problems, it delivers results within 500 seconds for 500 operations. Real-world job shops face frequent new job insertions, causing schedule instability; this study mitigates that by strategically incorporating priorities into rescheduling. BWSA-RL’s superior performance and ease of coding make it practical for industrial applications. While this study adopts three priority levels for simplicity, the algorithm can be extended to accommodate custom priority structures, enhancing flexibility for job shop managers. These features establish BWSA-RL as an effective metaheuristic for modern job shops.

## 5. Conclusions and future directions

This work focuses on the MODFJSP, incorporating new job arrivals as dynamic events. It aims to minimize makespan, total energy consumption, due-date penalties, and schedule instability. A three-level job priority system is introduced to improve due-date adherence. A reinforcement learning module integrates SARSA and Q-learning with a novel dynamic switching operator for better exploration-exploitation balance. Additionally, a hybrid crowding distance metric using cosine distances is introduced to improve diversity assessment. A population-based evolutionary algorithm, BWSA-RL, inspired by the black widow spiders, is developed to handle these challenges effectively.

Key contributions include a MILP model tested with IBM CPLEX for validation, an innovative conversion condition operator for dynamic RL switching, and an ablation study confirming the hybrid RL approach’s effectiveness. The proposed ADP objective is evaluated for job priorities, showing strong due-date adherence with minimal impact on makespan and energy consumption. The novel crowding distance metric outperforms existing approaches, and BWSA-RL is benchmarked against four state-of-the-art algorithms, demonstrating superior performance across key evaluation metrics. The rescheduling heuristic successfully minimizes instability.

Despite the promising results, this study has certain limitations that open avenues for future research. First, the experimental evaluation is conducted on synthetically generated benchmark instances; although these problems are widely used and carefully designed, validating the proposed approach on real industrial case studies would further strengthen its practical relevance. Second, the dynamic environment considered in this work is limited to new job insertions, while other common disruptions such as machine breakdowns, processing time variability, and priority changes remain unexplored. The comparison with exact optimization (CPLEX) is restricted to small-scale instances and single-objective formulations. As such, it does not fully reflect the complexity of the multi-objective dynamic scheduling problem addressed in this study. In addition, the reinforcement learning component is employed for adaptive parameter control rather than direct policy learning for scheduling decisions, which may limit its ability to fully exploit long-term learning potential. BWSA-RL employs use of copying and maintaining multiple populations during execution, this may result in higher computational cost for very large scale problems. Future studies may extend the proposed framework by incorporating additional dynamic events, investigating deep reinforcement learning for direct decision-making, and improving computational efficiency for large-scale industrial applications. Quantum-inspired reinforcement learning approaches, such as quantum policy learning, offer potential improvements in efficiency and stability, and represent a promising direction for future research in DFJSP.

## Supporting information

S1 Table Conversion condition operator analysis results of BWSA-RL for C-metric, GD and IGD. (DOCX)

S2 Table Comparison of performance of crowding distance operators for C-metric, GD and IGD. (DOCX)

S3 Table Due-dates non-conformance data for all five weight settings. (DOCX)

S4 Table Effect of each weight setting on optimization objectives. (DOCX)

S5 Table Comparison of RS1 and RS2 for optimization objectives and detailed statistical analysis. (DOCX)

S1 File ProblemSet. (ZIP)

S2 File SourceCode. (ZIP)
