# Supplementary material for: A novel multi-objective dynamic flexible job shop scheduling algorithm using reinforced learning based black widow spider algorithm
Source: PLoS One. 2026 Apr 20;21(4):e0347108. doi: 10.1371/journal.pone.0347108 (PMC13095024; doi:10.1371/journal.pone.0347108)
Supplement: S1 Table — (DOCX) [file pone.0347108.s001.docx]

S1 Table.

Data Table: Conversion condition operator analysis results of BWSA-RL for C-metric, GD and IGD

| **BWSA-RL (A) BWSA-Fixed (B) Vs BWSA-SLGA (C)** | | | | | | | | | | | | |
| --- | --- | --- | --- | --- | --- | --- | --- | --- | --- | --- | --- | --- |
|  |  | **C-Metric Values (Algorithm 1 vs Algorithm 2)** | | | | **GD metric values for A, B, & C** | | | **IGD metric values for A, B, & C** | | |  |
| **Problem** | **n x m (opr)** | **C(A,B)** | **C(B,A)** | **C(A,C)** | **C(C,A)** | **A (mean/std.)** | **B (mean/std.)** | **C (mean/std.)** | **A (mean/std.)** | **B (mean/std.)** | **C (mean/std.)** | **Win** |
| P01 | 5x3(25) | 0.467 | 0.342 | 0.553 | 0.221 | 0.0050/0.00309 | 0.0054/0.00286 | 0.0074/0.00537 | 0.0027/0.00098 | 0.0047/0.00216 | 0.0067/0.00291 | + |
| P02 | 5x5(25) | 0.57 | 0.282 | 0.663 | 0.22 | 0.0290/0.02022 | 0.0213/0.01337 | 0.0185/0.00917 | 0.0406/0.01112 | 0.0434/0.01267 | 0.0523/0.00939 | - |
| P03 | 8x5(40) | 0.716 | 0.184 | 0.775 | 0.128 | 0.0148/0.00512 | 0.0187/0.00759 | 0.0237/0.00558 | 0.0410/0.01044 | 0.0685/0.01109 | 0.0827/0.02628 | + |
| P04 | 8x5(40) | 0.776 | 0.097 | 0.803 | 0.086 | 0.0077/0.00404 | 0.0137/0.00468 | 0.0180/0.00807 | 0.0483/0.01331 | 0.0635/0.01739 | 0.0799/0.02508 | + |
| P05 | 10x5(50) | 0.619 | 0.238 | 0.641 | 0.174 | 0.0643/0.04798 | 0.0656/0.03578 | 0.0627/0.01913 | 0.0816/0.01421 | 0.1160/0.06481 | 0.1214/0.02170 | - |
| P06 | 10x8(50) | 0.8 | 0.058 | 0.851 | 0.117 | 0.0403/0.02790 | 0.0549/0.07569 | 0.0497/0.03831 | 0.1241/0.05737 | 0.1529/0.04922 | 0.1316/0.06003 | + |
| P07 | 10x10(50) | 0.784 | 0.08 | 0.7 | 0.166 | 0.0315/0.00867 | 0.0397/0.01124 | 0.0476/0.01467 | 0.1413/0.03228 | 0.1578/0.03511 | 0.1559/0.05022 | + |
| P08 | 15x5(75) | 0.673 | 0.268 | 0.832 | 0.124 | 0.0144/0.00344 | 0.0217/0.01051 | 0.0259/0.00623 | 0.0476/0.01149 | 0.0731/0.03050 | 0.0843/0.01507 | + |
| P09 | 15x10(75) | 0.74 | 0.146 | 0.74 | 0.135 | 0.0202/0.00537 | 0.0254/0.00844 | 0.0390/0.02001 | 0.0531/0.02242 | 0.0815/0.03185 | 0.0944/0.01779 | + |
| P10 | 15x15(75) | 0.622 | 0.264 | 0.885 | 0.071 | 0.0170/0.00452 | 0.0313/0.01471 | 0.0231/0.00592 | 0.0627/0.02982 | 0.1098/0.03803 | 0.1055/0.03447 | + |
| P11 | 20x5(160) | 0.817 | 0.107 | 0.938 | 0.041 | 0.0041/0.00164 | 0.0077/0.00309 | 0.0138/0.00355 | 0.0181/0.00493 | 0.0347/0.01530 | 0.0591/0.03084 | + |
| P12 | 20x6(160) | 0.84 | 0.102 | 0.882 | 0.058 | 0.0176/0.01166 | 0.0248/0.00862 | 0.0343/0.00887 | 0.0450/0.00604 | 0.0785/0.01300 | 0.1023/0.02560 | + |
| P13 | 20x8(160) | 0.713 | 0.151 | 0.713 | 0.112 | 0.0129/0.00920 | 0.0180/0.00726 | 0.0286/0.02001 | 0.0514/0.01510 | 0.0648/0.02438 | 0.0700/0.01429 | + |
| P14 | 20x10(160) | 0.758 | 0.109 | 0.99 | 0 | 0.0205/0.01277 | 0.0539/0.03044 | 0.0717/0.02239 | 0.1306/0.02939 | 0.1814/0.05947 | 0.1907/0.02758 | + |
| P15 | 25x5(200) | 0.841 | 0.062 | 0.806 | 0.045 | 0.0126/0.00810 | 0.0288/0.01440 | 0.0418/0.02680 | 0.0392/0.01270 | 0.0732/0.02405 | 0.1172/0.12288 | + |
| P16 | 25x8(200) | 0.779 | 0.103 | 0.881 | 0.077 | 0.0086/0.00245 | 0.0171/0.00638 | 0.0299/0.01830 | 0.0453/0.01593 | 0.0842/0.04337 | 0.0975/0.03398 | + |
| P17 | 25x10(200) | 0.913 | 0.012 | 0.87 | 0.071 | 0.0286/0.01595 | 0.0457/0.01598 | 0.0509/0.01136 | 0.0293/0.01158 | 0.0668/0.02474 | 0.0713/0.03911 | + |
| P18 | 30x5(240) | 0.745 | 0.154 | 0.923 | 0.043 | 0.0082/0.00452 | 0.0150/0.00724 | 0.0155/0.00590 | 0.0264/0.00605 | 0.0467/0.01011 | 0.0609/0.01685 | + |
| P19 | 30x8(240) | 0.702 | 0.122 | 0.875 | 0.116 | 0.0097/0.00438 | 0.0170/0.00665 | 0.0230/0.01238 | 0.0368/0.01316 | 0.0794/0.04653 | 0.0975/0.05393 | + |
| P20 | 30x10(240) | 0.83 | 0.084 | 0.871 | 0.041 | 0.0208/0.01217 | 0.0260/0.01248 | 0.0490/0.03781 | 0.0373/0.01089 | 0.0592/0.02668 | 0.1091/0.05056 | + |
| P21 | 35x5(350) | 0.813 | 0.115 | 0.825 | 0.014 | 0.0245/0.02221 | 0.0413/0.01674 | 0.0503/0.02592 | 0.0342/0.00864 | 0.1283/0.10566 | 0.1455/0.07712 | + |
| P22 | 35x10(350) | 0.806 | 0.102 | 0.812 | 0.044 | 0.0033/0.00075 | 0.0055/0.00140 | 0.0116/0.01044 | 0.0185/0.00420 | 0.0497/0.05285 | 0.0827/0.07314 | + |
| P23 | 35x15(350) | 0.448 | 0.108 | 0.617 | 0.071 | 0.0263/0.01616 | 0.0408/0.02252 | 0.0531/0.01585 | 0.0783/0.05196 | 0.1602/0.10444 | 0.1679/0.13963 | + |
| P24 | 40x10(400) | 0.744 | 0.126 | 0.83 | 0.054 | 0.0065/0.00253 | 0.0137/0.00598 | 0.0144/0.00738 | 0.0316/0.00792 | 0.1092/0.10734 | 0.1196/0.10378 | + |
| P25 | 40x15(400) | 0.778 | 0.099 | 0.748 | 0.077 | 0.0187/0.01629 | 0.0197/0.01596 | 0.0143/0.01171 | 0.0357/0.00666 | 0.0715/0.06187 | 0.0691/0.04227 | - |
| P26 | 40x20(400) | 0.792 | 0.147 | 0.811 | 0.117 | 0.0063/0.00165 | 0.0088/0.00274 | 0.0106/0.00321 | 0.0324/0.01062 | 0.0528/0.01906 | 0.0587/0.01335 | + |
| P27 | 50x10(500) | 0.909 | 0.059 | 0.738 | 0.043 | 0.0047/0.00144 | 0.0151/0.01284 | 0.0264/0.02544 | 0.0218/0.00709 | 0.0390/0.00709 | 0.0745/0.04130 | + |
| P28 | 50x15(500) | 0.778 | 0.081 | 0.82 | 0.064 | 0.0039/0.00141 | 0.0124/0.01117 | 0.0095/0.00948 | 0.0245/0.00558 | 0.0877/0.09208 | 0.0695/0.06438 | + |
| P29 | 50x18(500) | 0.639 | 0.13 | 0.628 | 0.087 | 0.0102/0.00720 | 0.0103/0.00686 | 0.0154/0.00818 | 0.0240/0.00483 | 0.1059/0.11744 | 0.1485/0.12950 | + |
| P30 | 50x20(500) | 0.811 | 0.099 | 0.71 | 0.039 | 0.0089/0.00801 | 0.0111/0.00712 | 0.0124/0.00884 | 0.0375/0.00806 | 0.0461/0.00721 | 0.1053/0.11053 | + |
| **Total wins 90%** | | | | | | | | | | | | |

The criterion for winning is that BWSA-RL must perform better in terms of all performance metrics, i.e. C-metric, GD and IGD.
