# Supplementary material for: A novel multi-objective dynamic flexible job shop scheduling algorithm using reinforced learning based black widow spider algorithm
Source: PLoS One. 2026 Apr 20;21(4):e0347108. doi: 10.1371/journal.pone.0347108 (PMC13095024; doi:10.1371/journal.pone.0347108)
Supplement: S2 Table — (DOCX) [file pone.0347108.s002.docx]

S2 Table.

Data Table: Comparison of performance of crowding distance operators for C-metric, GD and IGD

| **HCD (A) Vs MCDO (B) Vs HDED (C)** | | | | | | | | | | | | |
| --- | --- | --- | --- | --- | --- | --- | --- | --- | --- | --- | --- | --- |
|  |  | **C-Metric Values (Algorithm 1 vs Algorithm 2)** | | | | **GD metric values for A, B & C** | | | **IGD metric values for A, B & C** | | |  |
| **Problem** | **n x m (opr)** | **C(A,B)** | **C(B,A)** | **C(A,C)** | **C(C,A)** | **A (mean/std.)** | **B (mean/std.)** | **C (mean/std.)** | **A (mean/std.)** | **B (mean/std.)** | **C (mean/std.)** | **Win** |
| P01 | 5x3(25) | 0.782 | 0.788 | 0.78 | 0.811 | 0.0057/0.00126 | 0.0059/0.00106 | 0.0060/0.00116 | 0.0254/0.00754 | 0.0308/0.00769 | 0.0220/0.00698 | **-** |
| P02 | 5x5(25) | 0.816 | 0.638 | 0.653 | 0.791 | 0.0143/0.00343 | 0.0167/0.00243 | 0.0135/0.00411 | 0.0389/0.01170 | 0.0471/0.00876 | 0.0402/0.00857 | **-** |
| P03 | 8x5(40) | 0.803 | 0.501 | 0.773 | 0.55 | 0.0052/0.00102 | 0.0073/0.00210 | 0.0055/0.00091 | 0.0477/0.00620 | 0.0597/0.00672 | 0.0487/0.00582 | **+** |
| P04 | 8x5(40) | 0.805 | 0.507 | 0.742 | 0.593 | 0.0066/0.00313 | 0.0078/0.00316 | 0.0066/0.00257 | 0.0448/0.00590 | 0.0545/0.00806 | 0.0477/0.00612 | **+** |
| P05 | 10x5(50) | 0.859 | 0.551 | 0.833 | 0.546 | 0.0150/0.00336 | 0.0207/0.00488 | 0.0192/0.00523 | 0.0986/0.01646 | 0.1311/0.03584 | 0.1157/0.01881 | **+** |
| P06 | 10x8(50) | 0.584 | 0.233 | 0.699 | 0.115 | 0.0465/0.03413 | 0.0488/0.04665 | 0.0399/0.03681 | 0.2229/0.14573 | 0.2498/0.13771 | 0.2489/0.14522 | **-** |
| P07 | 10x10(50) | 0.838 | 0.163 | 0.874 | 0.16 | 0.0255/0.01495 | 0.0363/0.01859 | 0.0354/0.02502 | 0.0665/0.01503 | 0.0871/0.01511 | 0.0860/0.01628 | **+** |
| P08 | 15x5(75) | 0.934 | 0.659 | 0.91 | 0.693 | 0.0064/0.00157 | 0.0109/0.00138 | 0.0102/0.00115 | 0.0553/0.00552 | 0.0924/0.01064 | 0.0896/0.01006 | **+** |
| P09 | 15x10(75) | 0.936 | 0.243 | 0.927 | 0.289 | 0.0066/0.00266 | 0.0111/0.00249 | 0.0107/0.00370 | 0.0389/0.00768 | 0.0634/0.00928 | 0.0561/0.00710 | **+** |
| P10 | 15x15(75) | 0.828 | 0.161 | 0.836 | 0.176 | 0.0287/0.00946 | 0.0322/0.01053 | 0.0328/0.01087 | 0.0615/0.01929 | 0.0869/0.01548 | 0.0807/0.01260 | **+** |
| P11 | 20x5(160) | 0.96 | 0.45 | 0.93 | 0.441 | 0.0019/0.00034 | 0.0043/0.00058 | 0.0040/0.00063 | 0.0333/0.00507 | 0.0931/0.01674 | 0.1003/0.01734 | **+** |
| P12 | 20x6(160) | 0.953 | 0.393 | 0.969 | 0.393 | 0.0083/0.00178 | 0.0187/0.00285 | 0.0176/0.00347 | 0.0694/0.01016 | 0.1407/0.01859 | 0.1377/0.01568 | **+** |
| P13 | 20x8(160) | 0.959 | 0.436 | 0.945 | 0.504 | 0.0073/0.00134 | 0.0159/0.00281 | 0.0146/0.00198 | 0.0642/0.01057 | 0.1483/0.02125 | 0.1443/0.01817 | **+** |
| P14 | 20x10(160) | 0.979 | 0.219 | 0.98 | 0.191 | 0.0168/0.01193 | 0.0249/0.01293 | 0.0279/0.00945 | 0.0588/0.01119 | 0.1142/0.02330 | 0.1152/0.01699 | **+** |
| P15 | 25x5(200) | 0.981 | 0.553 | 0.959 | 0.572 | 0.0044/0.00173 | 0.0116/0.00184 | 0.0103/0.00125 | 0.0472/0.00626 | 0.1264/0.01547 | 0.1367/0.02120 | **+** |
| P16 | 25x8(200) | 0.975 | 0.26 | 0.959 | 0.237 | 0.0082/0.00183 | 0.0184/0.00229 | 0.0175/0.00182 | 0.0544/0.01140 | 0.1442/0.01780 | 0.1709/0.02133 | **+** |
| P17 | 25x10(200) | 0.976 | 0.302 | 0.953 | 0.315 | 0.0023/0.00045 | 0.0071/0.00130 | 0.0064/0.00141 | 0.0323/0.00569 | 0.1097/0.01891 | 0.1113/0.01380 | **+** |
| P18 | 30x5(240) | 0.974 | 0.465 | 0.956 | 0.49 | 0.0025/0.00038 | 0.0071/0.00079 | 0.0065/0.00061 | 0.0453/0.00839 | 0.1367/0.01540 | 0.1637/0.02713 | **+** |
| P19 | 30x8(240) | 0.987 | 0.313 | 0.973 | 0.305 | 0.0035/0.00082 | 0.0087/0.00123 | 0.0077/0.00064 | 0.0390/0.00697 | 0.1448/0.02034 | 0.1672/0.02039 | **+** |
| P20 | 30x10(240) | 0.978 | 0.235 | 0.984 | 0.262 | 0.0075/0.00317 | 0.0230/0.00604 | 0.0180/0.00335 | 0.0453/0.00841 | 0.1428/0.02100 | 0.1405/0.01360 | **+** |
| P21 | 35x5(350) | 0.8 | 0.745 | 0.796 | 0.769 | 0.0082/0.00127 | 0.0092/0.00133 | 0.0086/0.00117 | 0.0953/0.01281 | 0.1042/0.01490 | 0.1092/0.01610 | **+** |
| P22 | 35x10(350) | 0.872 | 0.49 | 0.681 | 0.454 | 0.0020/0.00031 | 0.0030/0.00037 | 0.0029/0.00042 | 0.0303/0.00371 | 0.0415/0.00440 | 0.0521/0.00761 | **+** |
| P23 | 35x15(350) | 0.876 | 0.504 | 0.846 | 0.556 | 0.0068/0.00144 | 0.0106/0.00164 | 0.0098/0.00114 | 0.0579/0.01389 | 0.0736/0.00874 | 0.0748/0.01024 | **+** |
| P24 | 40x10(400) | 0.92 | 0.331 | 0.84 | 0.308 | 0.0033/0.00045 | 0.0057/0.00054 | 0.0052/0.00045 | 0.0509/0.00750 | 0.0865/0.01137 | 0.0979/0.01354 | **+** |
| P25 | 40x15(400) | 0.922 | 0.441 | 0.89 | 0.436 | 0.0036/0.00064 | 0.0066/0.00092 | 0.0056/0.00075 | 0.0408/0.00527 | 0.0745/0.01209 | 0.0736/0.01107 | **+** |
| P26 | 40x20(400) | 0.753 | 0.604 | 0.634 | 0.485 | 0.0033/0.00071 | 0.0035/0.00062 | 0.0034/0.00057 | 0.0349/0.00495 | 0.0415/0.00666 | 0.0392/0.00558 | **+** |
| P27 | 50x10(500) | 0.882 | 0.71 | 0.798 | 0.781 | 0.0056/0.00124 | 0.0083/0.00177 | 0.0069/0.00110 | 0.0544/0.00920 | 0.0783/0.01231 | 0.0790/0.01428 | **+** |
| P28 | 50x15(500) | 0.878 | 0.529 | 0.83 | 0.519 | 0.0038/0.00055 | 0.0059/0.00078 | 0.0050/0.00060 | 0.0529/0.00562 | 0.0830/0.01341 | 0.0735/0.01114 | **+** |
| P29 | 50x18(500) | 0.892 | 0.291 | 0.75 | 0.311 | 0.0031/0.00115 | 0.0056/0.00153 | 0.0036/0.00054 | 0.0277/0.00404 | 0.0485/0.00648 | 0.0645/0.00761 | **+** |
| P30 | 50x20(500) | 0.923 | 0.385 | 0.847 | 0.403 | 0.0043/0.00080 | 0.0069/0.00147 | 0.0057/0.00060 | 0.0396/0.00478 | 0.0641/0.00820 | 0.0597/0.00567 | **+** |
| **Total wins 90%** | | | | | | | | | | | | |

The criterion for winning is that BWSA-RL must perform better in terms of all performance metrics, i.e. C-metric, GD and IGD.
