# Supplementary material for: A novel multi-objective dynamic flexible job shop scheduling algorithm using reinforced learning based black widow spider algorithm
Source: PLoS One. 2026 Apr 20;21(4):e0347108. doi: 10.1371/journal.pone.0347108 (PMC13095024; doi:10.1371/journal.pone.0347108)
Supplement: S3 Table — (DOCX) [file pone.0347108.s003.docx]

S3 Table.

Data Table: Due-dates non-conformance data for all five weight settings

|  | **Setting 1** | | | **Setting 2** | | | **Setting 3** | | | **Setting 4** | | | **Setting 5** | | |
| --- | --- | --- | --- | --- | --- | --- | --- | --- | --- | --- | --- | --- | --- | --- | --- |
|  | **W_H_=0.33, W_N_=0.33, W_L_=0.33** | | | **W_H_=0.5, W_N_=0.3, W_L_=0.2** | | | **W_H_=0.5, W_N_=0.4, W_L_=0.1** | | | **W_H_=0.6, W_N_=0.3, W_L_=0.1** | | | **W_H_=0.7, W_N_=0.2, W_L_=0.1** | | |
| **Prob-lem** | **NC_H_** | **NC_N_** | **NC_L_** | **NC_H_** | **NC_N_** | **NC_L_** | **NC_H_** | **NC_N_** | **NC_L_** | **NC_H_** | **NC_N_** | **NC_L_** | **NC_H_** | **NC_N_** | **NC_L_** |
| **P01** | 7.89 | 7.35 | 9.36 | 6.53 | 6.87 | 9.01 | 6.99 | 5.06 | 11.32 | 6.03 | 7.28 | 9.30 | 6.26 | 8.81 | 8.70 |
| **P02** | 0.22 | 0.60 | 1.49 | 0.07 | 0.56 | 1.24 | 0.07 | 0.17 | 2.02 | 0.12 | 0.34 | 2.02 | 0.00 | 0.28 | 1.48 |
| **P03** | 2.87 | 5.97 | 5.10 | 1.89 | 6.39 | 4.62 | 4.15 | 7.00 | 6.76 | 1.86 | 6.22 | 5.27 | 1.90 | 7.05 | 4.93 |
| **P04** | 5.12 | 6.13 | 2.16 | 4.40 | 6.86 | 3.93 | 3.14 | 2.88 | 8.56 | 1.85 | 5.95 | 8.22 | 0.95 | 9.11 | 9.43 |
| **P05** | 6.36 | 6.54 | 4.90 | 3.84 | 5.44 | 8.32 | 4.50 | 5.17 | 8.82 | 1.99 | 5.44 | 7.89 | 1.47 | 7.01 | 8.17 |
| **P06** | 0.00 | 0.00 | 0.10 | 0.30 | 0.00 | 0.50 | 0.25 | 0.00 | 0.17 | 0.00 | 0.00 | 0.50 | 0.00 | 0.00 | 0.13 |
| **P07** | 0.00 | 0.08 | 0.08 | 0.00 | 0.50 | 0.09 | 0.13 | 0.25 | 0.31 | 0.04 | 0.00 | 0.36 | 0.00 | 0.17 | 0.32 |
| **P08** | 20.76 | 18.96 | 25.57 | 11.31 | 20.69 | 31.19 | 10.20 | 17.98 | 33.51 | 8.87 | 18.57 | 32.73 | 6.67 | 18.51 | 32.95 |
| **P09** | 1.20 | 1.97 | 3.22 | 0.59 | 1.38 | 3.72 | 0.71 | 1.20 | 3.97 | 0.52 | 1.26 | 4.79 | 0.31 | 1.58 | 3.64 |
| **P10** | 0.00 | 0.27 | 0.00 | 0.05 | 0.05 | 0.00 | 0.00 | 0.29 | 0.00 | 0.06 | 0.50 | 0.00 | 0.00 | 0.27 | 0.00 |
| **P11** | 44.03 | 42.01 | 49.11 | 35.01 | 42.53 | 55.24 | 35.83 | 34.47 | 62.07 | 26.40 | 46.90 | 62.31 | 21.04 | 51.47 | 62.88 |
| **P12** | 15.33 | 16.31 | 21.63 | 10.01 | 18.66 | 22.70 | 11.39 | 13.27 | 26.89 | 7.09 | 16.14 | 25.21 | 5.23 | 18.52 | 24.76 |
| **P13** | 10.96 | 10.65 | 11.14 | 9.07 | 11.96 | 13.94 | 9.78 | 9.99 | 16.32 | 8.97 | 10.62 | 15.45 | 7.61 | 13.98 | 14.93 |
| **P14** | 6.58 | 6.79 | 8.69 | 2.80 | 5.97 | 8.18 | 3.84 | 3.39 | 8.36 | 5.27 | 7.03 | 10.17 | 2.84 | 6.53 | 10.03 |
| **P15** | 64.27 | 59.09 | 58.15 | 34.80 | 67.39 | 71.80 | 29.12 | 59.53 | 78.69 | 23.66 | 74.35 | 76.49 | 25.70 | 70.52 | 76.94 |
| **P16** | 30.41 | 26.61 | 28.30 | 26.00 | 29.93 | 37.06 | 23.79 | 24.46 | 37.99 | 22.75 | 27.24 | 37.15 | 15.84 | 32.63 | 36.09 |
| **P17** | 32.20 | 33.59 | 33.49 | 23.69 | 31.91 | 40.26 | 25.10 | 27.93 | 42.68 | 24.03 | 31.84 | 44.41 | 19.33 | 38.74 | 43.96 |
| **P18** | 114.8 | 116.0 | 111.6 | 92.39 | 120.4 | 145.9 | 98.93 | 113.9 | 153.3 | 76.62 | 128.6 | 151.0 | 75.20 | 137.3 | 150.1 |
| **P19** | 58.67 | 52.59 | 60.59 | 46.72 | 57.37 | 69.75 | 45.78 | 49.38 | 68.48 | 38.14 | 60.46 | 72.73 | 38.28 | 61.13 | 70.70 |
| **P20** | 17.71 | 17.28 | 13.67 | 14.05 | 16.00 | 16.46 | 13.73 | 13.20 | 18.92 | 12.77 | 15.46 | 18.03 | 8.96 | 17.74 | 20.71 |
| **P21** | 134.8 | 136.3 | 145.0 | 106.9 | 144.2 | 158.4 | 110.5 | 137.5 | 169.4 | 83.93 | 156.1 | 165.0 | 88.86 | 166.2 | 162.5 |
| **P22** | 75.78 | 77.10 | 77.81 | 68.97 | 83.72 | 92.08 | 68.61 | 78.02 | 95.59 | 71.23 | 85.10 | 100.7 | 49.84 | 87.51 | 94.70 |
| **P23** | 14.47 | 13.98 | 14.89 | 11.20 | 14.27 | 15.85 | 12.46 | 13.87 | 18.69 | 9.32 | 12.27 | 17.35 | 9.24 | 14.49 | 18.96 |
| **P24** | 79.66 | 96.07 | 95.71 | 69.45 | 96.98 | 102.5 | 87.52 | 97.11 | 115.4 | 65.16 | 100.1 | 110.2 | 57.59 | 101.3 | 108.5 |
| **P25** | 23.55 | 22.97 | 25.14 | 19.16 | 22.26 | 29.80 | 19.17 | 20.18 | 29.81 | 17.64 | 22.88 | 32.56 | 17.49 | 25.38 | 30.15 |
| **P26** | 32.91 | 29.93 | 33.27 | 31.67 | 34.71 | 38.13 | 30.70 | 32.39 | 40.28 | 34.03 | 37.09 | 44.14 | 27.11 | 37.40 | 41.55 |
| **P27** | 55.53 | 48.59 | 53.98 | 45.41 | 50.96 | 57.58 | 46.73 | 45.45 | 63.01 | 40.70 | 51.29 | 65.47 | 45.23 | 54.03 | 63.01 |
| **P28** | 41.66 | 43.99 | 38.99 | 35.21 | 41.96 | 42.83 | 35.20 | 40.95 | 45.66 | 32.89 | 44.43 | 45.84 | 27.00 | 43.26 | 40.70 |
| **P29** | 56.68 | 53.58 | 61.39 | 58.30 | 64.67 | 71.89 | 57.46 | 63.36 | 76.69 | 53.24 | 62.10 | 75.35 | 51.06 | 67.00 | 74.63 |
| **P30** | 17.20 | 20.79 | 19.07 | 15.39 | 21.74 | 21.44 | 15.44 | 19.41 | 21.46 | 14.63 | 20.48 | 22.67 | 12.96 | 22.13 | 21.76 |
| **Avg** | **32.4** | **32.4** | **33.8** | **26.2** | **34.2** | **39.1** | **27.0** | **31.3** | **42.2** | **23.0** | **35.2** | **42.1** | **20.8** | **37.3** | **41.2** |
