# Supplementary material for: A novel multi-objective dynamic flexible job shop scheduling algorithm using reinforced learning based black widow spider algorithm
Source: PLoS One. 2026 Apr 20;21(4):e0347108. doi: 10.1371/journal.pone.0347108 (PMC13095024; doi:10.1371/journal.pone.0347108)
Supplement: S4 Table — (DOCX) [file pone.0347108.s004.docx]

S4 Table.

Data Table: Effect of each weight setting on optimization objectives

|  | **Setting 1** | | | **Setting 2** | | | **Setting 3** | | | **Setting 4** | | | **Setting 5** | | |
| --- | --- | --- | --- | --- | --- | --- | --- | --- | --- | --- | --- | --- | --- | --- | --- |
|  | **W_H_=0.33, W_N_=0.33, W_L_=0.33** | | | **W_H_=0.5, W_N_=0.3, W_L_=0.2** | | | **W_H_=0.5, W_N_=0.4, W_L_=0.1** | | | **W_H_=0.6, W_N_=0.3, W_L_=0.1** | | | **W_H_=0.7, W_N_=0.2, W_L_=0.1** | | |
| **Prob-lem** | **MK** | **TEC** | **ADP** | **MK** | **TEC** | **ADP** | **MK** | **TEC** | **ADP** | **MK** | **TEC** | **ADP** | **MK** | **TEC** | **ADP** |
| **P01** | 62.0 | 101.1 | 3.2 | 62.5 | 100.8 | 3.2 | 64.9 | 100.9 | 3.1 | 64.3 | 100.6 | 3.3 | 62.5 | 101.0 | 3.2 |
| **P02** | 38.8 | 72.2 | 0.3 | 40.5 | 72.2 | 0.3 | 40.1 | 72.1 | 0.2 | 39.6 | 72.3 | 0.3 | 38.9 | 72.4 | 0.1 |
| **P03** | 58.4 | 148.8 | 1.6 | 59.0 | 148.7 | 1.5 | 58.8 | 148.7 | 1.3 | 61.7 | 148.5 | 1.8 | 59.2 | 148.5 | 1.1 |
| **P04** | 57.7 | 148.8 | 1.5 | 58.2 | 148.6 | 1.5 | 58.6 | 149.1 | 1.5 | 62.2 | 148.7 | 1.4 | 61.0 | 148.6 | 1.2 |
| **P05** | 53.8 | 137.6 | 1.8 | 53.4 | 138.1 | 1.2 | 54.2 | 137.7 | 1.2 | 54.7 | 137.7 | 1.6 | 54.2 | 138.1 | 1.3 |
| **P06** | 33.0 | 123.1 | 0.1 | 32.3 | 122.4 | 0.0 | 31.9 | 123.3 | 0.0 | 32.1 | 122.4 | 0.0 | 33.1 | 122.4 | 0.0 |
| **P07** | 30.4 | 105.6 | 0.1 | 29.6 | 106.6 | 0.0 | 32.5 | 106.0 | 0.1 | 29.4 | 106.3 | 0.0 | 30.5 | 106.2 | 0.0 |
| **P08** | 85.0 | 233.8 | 7.8 | 86.9 | 233.6 | 6.3 | 86.7 | 233.7 | 5.8 | 86.3 | 233.9 | 4.6 | 85.3 | 234.2 | 4.1 |
| **P09** | 44.2 | 190.0 | 0.6 | 41.2 | 190.9 | 0.3 | 43.3 | 190.2 | 0.3 | 42.5 | 191.0 | 0.2 | 44.2 | 190.0 | 0.4 |
| **P10** | 26.9 | 120.7 | 0.0 | 25.8 | 120.9 | 0.0 | 26.3 | 121.1 | 0.0 | 25.6 | 121.2 | 0.0 | 26.8 | 121.6 | 0.0 |
| **P11** | 136.0 | 343.6 | 16.2 | 135.4 | 343.7 | 14.8 | 135.5 | 344.0 | 13.5 | 135.9 | 343.7 | 12.5 | 138.0 | 343.1 | 10.6 |
| **P12** | 82.3 | 295.9 | 5.7 | 81.4 | 296.0 | 5.0 | 81.5 | 296.1 | 4.3 | 81.4 | 296.2 | 4.0 | 81.7 | 296.2 | 3.1 |
| **P13** | 70.7 | 306.8 | 3.8 | 69.6 | 307.9 | 3.3 | 69.6 | 308.2 | 2.8 | 71.3 | 307.3 | 2.7 | 70.9 | 308.3 | 2.4 |
| **P14** | 76.5 | 373.9 | 1.6 | 78.8 | 373.3 | 1.8 | 76.8 | 373.7 | 1.3 | 78.4 | 373.7 | 1.2 | 78.2 | 373.5 | 1.4 |
| **P15** | 154.1 | 529.8 | 20.1 | 154.4 | 530.0 | 17.3 | 156.6 | 530.5 | 17.3 | 157.1 | 530.1 | 13.7 | 155.8 | 529.5 | 11.0 |
| **P16** | 103.3 | 414.7 | 9.8 | 103.7 | 415.5 | 8.9 | 102.5 | 416.1 | 8.4 | 108.3 | 414.5 | 7.7 | 104.8 | 414.5 | 6.0 |
| **P17** | 113.9 | 530.5 | 11.1 | 109.4 | 536.9 | 9.0 | 112.0 | 534.9 | 8.5 | 115.6 | 535.7 | 8.9 | 113.2 | 534.2 | 6.9 |
| **P18** | 260.2 | 959.6 | 40.1 | 260.4 | 960.9 | 36.1 | 266.0 | 959.4 | 37.6 | 262.0 | 960.2 | 32.5 | 265.2 | 959.9 | 31.8 |
| **P19** | 153.1 | 747.3 | 19.8 | 151.8 | 747.9 | 17.6 | 151.4 | 748.3 | 15.5 | 150.9 | 749.1 | 14.8 | 151.1 | 749.9 | 12.5 |
| **P20** | 86.8 | 481.0 | 4.6 | 85.2 | 481.3 | 3.8 | 90.6 | 479.2 | 3.8 | 88.4 | 480.4 | 4.0 | 89.5 | 479.6 | 3.9 |
| **P21** | 281.3 | 806.9 | 48.9 | 280.2 | 807.1 | 49.1 | 276.9 | 806.8 | 44.8 | 280.5 | 807.0 | 44.7 | 279.1 | 807.7 | 36.7 |
| **P22** | 219.1 | 1060.8 | 28.4 | 219.7 | 1060.1 | 26.2 | 214.2 | 1062.8 | 23.3 | 214.2 | 1063.2 | 22.5 | 212.0 | 1063.7 | 19.6 |
| **P23** | 90.1 | 734.6 | 4.1 | 89.2 | 735.0 | 3.9 | 89.9 | 735.3 | 3.4 | 93.3 | 734.0 | 4.0 | 90.8 | 733.7 | 3.1 |
| **P24** | 216.0 | 1014.2 | 32.1 | 220.8 | 1012.6 | 32.1 | 220.5 | 1011.8 | 29.9 | 217.5 | 1012.3 | 26.4 | 220.5 | 1010.7 | 25.4 |
| **P25** | 110.6 | 824.8 | 7.4 | 114.0 | 819.8 | 7.8 | 111.2 | 821.6 | 5.7 | 109.3 | 822.6 | 5.2 | 112.6 | 821.1 | 5.5 |
| **P26** | 148.9 | 1140.4 | 10.9 | 156.7 | 1136.5 | 12.1 | 152.4 | 1135.8 | 10.6 | 144.3 | 1144.4 | 8.8 | 152.7 | 1138.8 | 9.9 |
| **P27** | 149.4 | 881.4 | 17.3 | 146.4 | 880.7 | 15.7 | 149.0 | 880.9 | 14.2 | 149.6 | 881.1 | 14.1 | 147.1 | 879.1 | 11.9 |
| **P28** | 143.9 | 1057.0 | 12.3 | 145.3 | 1055.3 | 12.1 | 146.4 | 1052.8 | 11.4 | 146.8 | 1053.2 | 11.9 | 148.1 | 1052.4 | 11.3 |
| **P29** | 173.9 | 1307.7 | 19.7 | 181.2 | 1303.6 | 19.6 | 177.7 | 1303.3 | 18.4 | 179.2 | 1306.5 | 18.9 | 188.6 | 1302.6 | 19.4 |
| **P30** | 107.1 | 1052.4 | 4.9 | 117.9 | 1048.7 | 5.8 | 111.5 | 1052.1 | 4.9 | 111.0 | 1051.7 | 4.4 | 111.1 | 1050.3 | 4.1 |
| **Avg** | **112.2** | **541.5** | **11.2** | **113.0** | **541.2** | **10.5** | **113.0** | **541.2** | **9.8** | **113.1** | **541.6** | **9.2** | **113.6** | **541.1** | **8.3** |
