# Supplementary material for: A novel multi-objective dynamic flexible job shop scheduling algorithm using reinforced learning based black widow spider algorithm
Source: PLoS One. 2026 Apr 20;21(4):e0347108. doi: 10.1371/journal.pone.0347108 (PMC13095024; doi:10.1371/journal.pone.0347108)
Supplement: S5 Table — (DOCX) [file pone.0347108.s005.docx]

**S5 Table.**

**Data Table: Comparison of RS1 and RS2 for optimization objectives and detailed statistical analysis**

| **Problem** | **Size** | **Average MK** | | **Average TEC** | | **Average ADP** | | **Average INS** | |
| --- | --- | --- | --- | --- | --- | --- | --- | --- | --- |
|  |  | **RS1** | **RS2** | **RS1** | **RS2** | **RS1** | **RS2** | **RS1** | **RS2** |
| P01 | 5x3(25) | 96.648 | 105.547 | 80.305 | 83.448 | 5.312 | 7.247 | 0.373 | 0.2704 |
| P02 | 5x5(25) | 58.530 | 62.546 | 41.951 | 43.308 | 0.837 | 0.732 | 0.365 | 0.0000 |
| P03 | 8x5(40) | 75.995 | 78.200 | 72.143 | 85.968 | 1.458 | 2.234 | 0.307 | 0.0000 |
| P04 | 8x5(40) | 72.594 | 78.250 | 67.243 | 64.052 | 1.972 | 2.243 | 0.292 | 0.0431 |
| P05 | 10x5(50) | 72.043 | 71.897 | 57.655 | 56.843 | 1.775 | 2.278 | 0.310 | 0.3213 |
| P06 | 10x8(50) | 41.860 | 43.800 | 32.035 | 35.631 | 0.056 | 0.095 | 0.264 | 0.0000 |
| P07 | 10x10(50) | 39.501 | 42.375 | 39.017 | 41.229 | 0.096 | 0.079 | 0.240 | 0.0969 |
| P08 | 15x5(75) | 90.239 | 94.300 | 83.793 | 83.332 | 4.832 | 5.653 | 0.335 | 0.0000 |
| P09 | 15x10(75) | 50.553 | 55.018 | 25.517 | 30.438 | 0.616 | 0.578 | 0.260 | 0.0213 |
| P10 | 15x15(75) | 30.412 | 36.552 | 41.809 | 50.799 | 0.002 | 0.022 | 0.186 | 0.0344 |
| P11 | 20x5(160) | 143.586 | 156.102 | 241.445 | 248.924 | 12.524 | 17.318 | 0.508 | 0.0472 |
| P12 | 20x6(160) | 92.597 | 90.162 | 131.577 | 117.449 | 4.754 | 4.469 | 0.406 | 0.3821 |
| P13 | 20x8(160) | 85.011 | 91.987 | 100.726 | 104.665 | 2.327 | 3.135 | 0.364 | 0.0855 |
| P14 | 20x10(160) | 92.009 | 92.344 | 148.536 | 149.202 | 1.617 | 1.975 | 0.416 | 0.1019 |
| P15 | 25x5(200) | 176.989 | 177.785 | 221.995 | 219.092 | 13.668 | 17.100 | 0.493 | 0.1034 |
| P16 | 25x8(200) | 115.913 | 117.674 | 29.978 | 26.543 | 5.198 | 5.157 | 0.318 | 0.1169 |
| P17 | 25x10(200) | 119.378 | 119.364 | 46.244 | 37.385 | 6.064 | 6.318 | 0.306 | 0.0869 |
| P18 | 30x5(240) | 285.830 | 286.288 | 667.697 | 664.284 | 26.859 | 28.394 | 0.552 | 0.5312 |
| P19 | 30x8(240) | 161.399 | 167.321 | 242.551 | 254.448 | 12.263 | 15.167 | 0.528 | 0.1053 |
| P20 | 30x10(240) | 101.025 | 104.281 | 235.715 | 242.237 | 3.683 | 4.584 | 0.520 | 0.0746 |
| P21 | 35x5(350) | 310.230 | 318.005 | 601.241 | 578.176 | 34.218 | 32.041 | 0.589 | 0.1492 |
| P22 | 35x10(350) | 223.659 | 223.652 | 627.519 | 584.985 | 17.754 | 21.105 | 0.656 | 0.1084 |
| P23 | 35x15(350) | 113.261 | 105.196 | 442.722 | 368.787 | 4.625 | 3.925 | 0.690 | 0.1365 |
| P24 | 40x10(400) | 217.201 | 229.100 | 564.406 | 585.581 | 20.634 | 28.083 | 0.656 | 0.0000 |
| P25 | 40x15(400) | 128.034 | 120.444 | 114.620 | 62.499 | 5.211 | 4.333 | 0.449 | 0.0941 |
| P26 | 40x20(400) | 140.024 | 150.200 | 237.959 | 260.195 | 4.849 | 6.696 | 0.514 | 0.0000 |
| P27 | 50x10(500) | 187.770 | 196.885 | 461.493 | 478.908 | 15.399 | 17.491 | 0.651 | 0.1112 |
| P28 | 50x15(500) | 171.398 | 164.843 | 438.667 | 353.808 | 11.001 | 10.443 | 0.666 | 0.1312 |
| P29 | 50x18(500) | 176.546 | 322.000 | 586.012 | 618.545 | 13.662 | 25.296 | 0.715 | 0.0000 |
| P30 | 50x20(500) | 140.945 | 137.311 | 845.848 | 743.924 | 7.749 | 6.502 | 0.763 | 0.126 |

**Statistical analysis of MK for RS1 & RS2**

| *Source of Variation* | *SS* | *df* | *MS* | *F* | *P-value* | *F crit* |
| --- | --- | --- | --- | --- | --- | --- |
| Between Groups | 868.2934 | 1 | 868.2934 | 0.159699 | 0.690902 | 4.006873 |
| Within Groups | 315349.4 | 58 | 5437.058 |  |  |  |
| Total | 316217.7 | 59 |  |  |  |  |

**Statistical analysis of TEC for RS1 & RS2**

| *Source of Variation* | *SS* | *df* | *MS* | *F* | *P-value* | *F crit* |
| --- | --- | --- | --- | --- | --- | --- |
| Between Groups | 1073.016 | 1 | 1073.016 | 0.019555 | 0.88927 | 4.006873 |
| Within Groups | 3182490 | 58 | 54870.52 |  |  |  |
| Total | 3183563 | 59 |  |  |  |  |

**Statistical analysis of ADP for RS1 & RS2**

| *Source of Variation* | *SS* | *df* | *MS* | *F* | *P-value* | *F crit* |
| --- | --- | --- | --- | --- | --- | --- |
| Between Groups | 26.23906 | 1 | 26.23906 | 0.323891 | 0.571476 | 4.006873 |
| Within Groups | 4698.697 | 58 | 81.01202 |  |  |  |
| Total | 4724.936 | 59 |  |  |  |  |

**Statistical analysis of INS for RS1 & RS2**

| *Source of Variation* | *SS* | *df* | *MS* | *F* | *P-value* | *F crit* |
| --- | --- | --- | --- | --- | --- | --- |
| Between Groups | 1.807176 | 1 | 1.807176 | 86.94867 | 3.88E-13 | 4.006873 |
| Within Groups | 1.205495 | 58 | 0.020784 |  |  |  |
| Total | 3.012671 | 59 |  |  |  |  |
